# Involvement Of Vascular Aldosterone Synthase In Phosphate-Induced Osteogenic Transformation Of Vascular Smooth Muscle Cells

**DOI:** 10.1038/s41598-017-01882-2

**Published:** 2017-05-17

**Authors:** Ioana Alesutan, Jakob Voelkl, Martina Feger, Denise V. Kratschmar, Tatsiana Castor, Sobuj Mia, Michael Sacherer, Robert Viereck, Oliver Borst, Christina Leibrock, Meinrad Gawaz, Makoto Kuro-o, Stefan Pilz, Andreas Tomaschitz, Alex Odermatt, Burkert Pieske, Carsten A. Wagner, Florian Lang

**Affiliations:** 1 Department of Physiology, University of Tübingen, Tübingen, Germany; 2 Department of Internal Medicine and Cardiology, Charité University Medicine, Campus Virchow-Klinikum, Berlin, Germany; 3 Berlin Institute of Health (BIH), Berlin, Germany; 4 Department of Pharmaceutical Sciences, and the National Center for Excellence in Research NCCR Kidney.CH, University of Basel, Basel, Switzerland; 5 Div. of Cardiology, Medical University of Graz and Ludwig Boltzmann Institute for Translational Heart Failure Research, Graz, Austria; 6 Department of Cardiology and Cardiovascular Medicine, University of Tübingen, Tübingen, Germany; 7 Center for Molecular Medicine, Jichi Medical University, Shimotsuke, Japan; 8 Department of Internal Medicine, Division of Endocrinology and Metabolism, Medical University of Graz, Graz, Austria; 9 Bad Gleichenberg Clinic, Bad Gleichenberg, Austria; 10 Department of Cardiology, University of Graz, Graz, Austria; Department of Internal Medicine and Cardiology, German Heart Center Berlin (DHZB), Berlin, Germany; 11 Institute of Physiology, University of Zurich, and the National Center for Excellence in Research NCCR Kidney, Zurich, Switzerland

## Abstract

Vascular calcification resulting from hyperphosphatemia is a major determinant of mortality in chronic kidney disease (CKD). Vascular calcification is driven by aldosterone-sensitive osteogenic transformation of vascular smooth muscle cells (VSMCs). We show that even in absence of exogenous aldosterone, silencing and pharmacological inhibition (spironolactone, eplerenone) of the mineralocorticoid receptor (MR) ameliorated phosphate-induced osteo-/chondrogenic transformation of primary human aortic smooth muscle cells (HAoSMCs). High phosphate concentrations up-regulated aldosterone synthase (CYP11B2) expression in HAoSMCs. Silencing and deficiency of CYP11B2 in VSMCs ameliorated phosphate-induced osteogenic reprogramming and calcification. Phosphate treatment was followed by nuclear export of APEX1, a CYP11B2 transcriptional repressor. APEX1 silencing up-regulated *CYP11B2* expression and stimulated osteo-/chondrogenic transformation. APEX1 overexpression blunted the phosphate-induced osteo-/chondrogenic transformation and calcification of HAoSMCs. *Cyp11b2* expression was higher in aortic tissue of hyperphosphatemic klotho-hypomorphic (*kl/kl*) mice than in wild-type mice. In adrenalectomized *kl/kl* mice, spironolactone treatment still significantly ameliorated aortic osteoinductive reprogramming. Our findings suggest that VSMCs express aldosterone synthase, which is up-regulated by phosphate-induced disruption of APEX1-dependent gene suppression. Vascular CYP11B2 may contribute to stimulation of VSMCs osteo-/chondrogenic transformation during hyperphosphatemia.

## Introduction

Vascular calcification with deposition of calcium-phosphate increases the risk of cardiovascular events in aging, diabetes and chronic kidney disease (CKD)^1^. Vascular calcification is therefore a powerful predictor of cardiovascular and all-cause mortality^2^. The impaired renal phosphate elimination in CKD patients increases extracellular phosphate concentration, which predisposes to calcification of the medial artery layer^3^. Plasma phosphate levels, even within the normal range, are predictive of cardiovascular events, heart failure and death^4, 5^.

Vascular calcification is an active process, promoted by vascular smooth muscle cells (VSMCs)^6^. In response to elevated extracellular phosphate concentrations, VSMCs differentiate and undergo osteo-/chondrogenic reprogramming^7^. This reprogramming involves enhanced expression of type III sodium-dependent phosphate transporter PiT1 (SLC20A1)^8^ and is characterized by expression of osteoblastic transcription factors Msh homeobox 2 (MSX2) and core-binding factor alpha 1 (CBFA1, encoded by the runt-related transcription factor 2; RUNX2 gene)^9, 10^. Inhibition of CBFA1 ameliorates vascular calcification^11^. Osteo-/chondrogenic reprogramming leads to expression of tissue-nonspecific alkaline phosphatase (ALPL), which hydrolyses the endogenous calcification inhibitor pyrophosphate, and which is therefore crucial in the formation of vascular calcification^1^. The markers of vascular osteo-/chondrogenic transformation are up-regulated before the onset of vascular calcification^12^. Indicators of VSMC osteo-/chondrogenic transformation are observed in vessels from human CKD patients^13^. Vessels from dialysis patients are more prone to medial calcification than vessels from healthy individuals *ex vivo* ^14^. Therefore, the osteoinductive alterations of vascular tissue in CKD predisposes to vascular calcification^14^. However, the complex events at the onset of the signalling cascade leading to phenotypic transformation of VSMCs are still incompletely understood^4^.

VSMCs express the mineralocorticoid receptor (MR)^15^. Stimulation of the MR by aldosterone triggers osteoinductive signaling^15–20^ by up-regulation of PIT1 expression^20, 21^. The PIT1 promoter sequence harbours putative MR response elements and PIT1 is required for aldosterone-induced osteogenic remodelling^20^. Hyperaldosteronism may contribute to vascular calcification in hyperphosphatemic klotho-hypomorphic (*kl/kl*) mice^22^. The hyperaldosteronism of CKD thus presumably contributes to the triggering of osteo-/chondrogenic signaling^23^. Accordingly, treatment with the MR antagonist spironolactone reduces cardio-/cerebrovascular mortality in dialysis patients^24^. Further trials are being conducted to investigate the potential benefits of spironolactone in CKD^25, 26^. The beneficial effects of spironolactone in renal disease may be independent of elevated circulating aldosterone levels^27, 28^.

Aldosterone synthesis may not be restricted to adrenal glands but aldosterone may be locally produced in several tissues^29^. Extra-adrenal steroid hormone synthesis was first reported in brain tissue^30^, and was later discussed for cardiac^31^, renal^32^ and vascular tissues^33^. Vascular cells, especially endothelial cells, are able to express the aldosterone synthase (also known as cytochrome P450 family 11 subfamily B member 2 or CYP11B2) and may therefore produce aldosterone^33–35^. CYP11B2 is localized to the mitochondrial inner membrane and has steroid 18-hydroxylase activity to synthesize aldosterone as well as 18-hydroxycorticosterone and steroid 11 beta-hydroxylase activity^36, 37^. However, the physiological relevance of vascular-derived aldosterone remains controversial, due to very low levels of aldosterone produced by VSMCs^38^. Nonetheless, in human atheroma-plaques, CYP11B2 expression is up-regulated^39^ and expression of CYP11B2 in VSMCs contributes to reactive oxygen species generation^40^. Vascular CYP11B2-dependent production of aldosterone or another yet to be identified MR ligand may be relevant in pathological conditions^31^.

The present study thus explored the possibility that vascular CYP11B2 may participate in the induction of osteo-/chondrogenic transformation of VSMCs.

## Results

### Effects of mineralocorticoid receptor blockade on phosphate-induced vascular calcification *in vitro*

**Figure 1:**
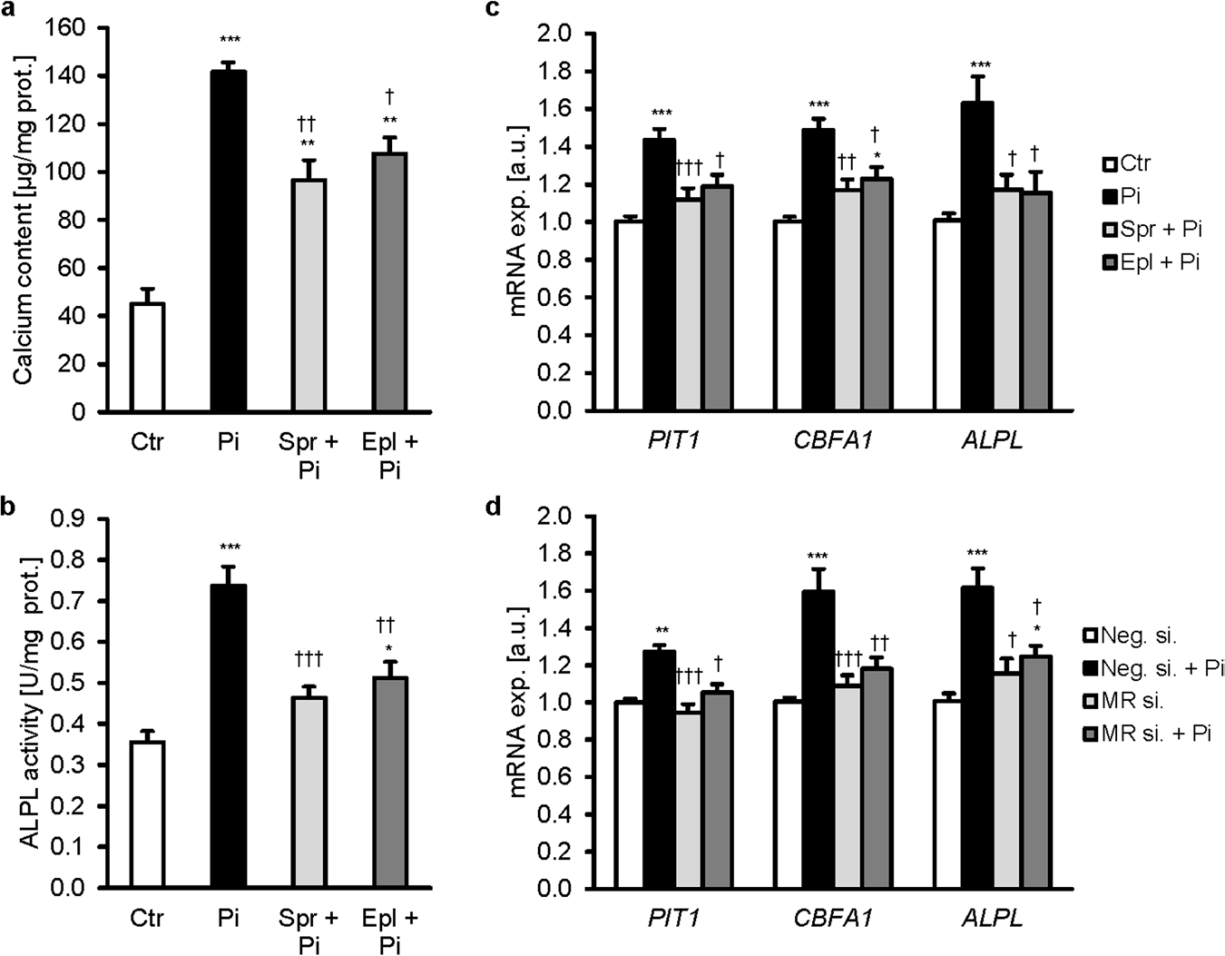
Mineralocorticoid receptor blockade ameliorates phosphate-induced calcification *in vitro*. Arithmetic means ± SEM of calcium content (**a**, n = 3; µg/mg protein), alkaline phosphatase activity (**b**, n = 6; units/mg protein) and of *PIT1*, *CBFA1* and *ALPL* relative mRNA expression (**c**, n = 14; arbitrary units, a.u.) in HAoSMCs following treatment with (Pi) or without (Ctr) phosphate and with or without additional treatment with 10 µM spironolactone (Spr) or 10 µM eplerenone (Epl). (**d**) Arithmetic means ± SEM of *PIT1*, *CBFA1* and *ALPL* relative mRNA expression (n = 10; a.u.) in HAoSMCs following silencing for 48 hours with negative control siRNA (Neg.si.) or MR siRNA (MRsi.) without or with treatment for 24 hours with phosphate (Pi). *(p < 0.05), **(p < 0.01), ***(p < 0.001) statistically significant vs control treated or Neg.si. silenced HAoSMCs, respectively. ^†^(p < 0.05), ^††^(p < 0.01), ^†††^(p < 0.001) statistically significant vs Pi treated or Neg.si. silenced and Pi treated HAoSMCs, respectively.

To elucidate the effects of MR blockade under elevated phosphate conditions, HAoSMCs were treated with phosphate in the absence or presence of the MR antagonists spironolactone and eplerenone, in charcoal-stripped FBS media. Treatment of HAoSMCs with phosphate increased calcium deposition (Fig. 1a), alkaline phosphatase activity (Fig. 1b) as well as *PiT1* and osteogenic and chondrogenic markers *CBFA1*, *ALPL, SOX9* and osterix (*SP7*) mRNA expression (Fig. 1c; Supplementary Fig. S1). These effects were significantly blunted in the presence of spironolactone or eplerenone. To further investigate the role of MR in VSMC osteo-/chondrogenic transformation, the MR gene (NR3C2) was silenced in HAoSMCs (Supplementary Fig. S2). *PiT1*, *CBFA1* and *ALPL* mRNA levels were up-regulated by phosphate in negative control silenced HAoSMCs, but not in HAoSMCs silenced with MR siRNA (Fig. 1d). Thus, MR blockade ameliorated phosphate-induced osteo-/chondrogenic transformation of HAoSMCs even in the absence of exogenous aldosterone.

### Regulation of aldosterone synthase expression by phosphate in VSMCs

**Figure 2:**
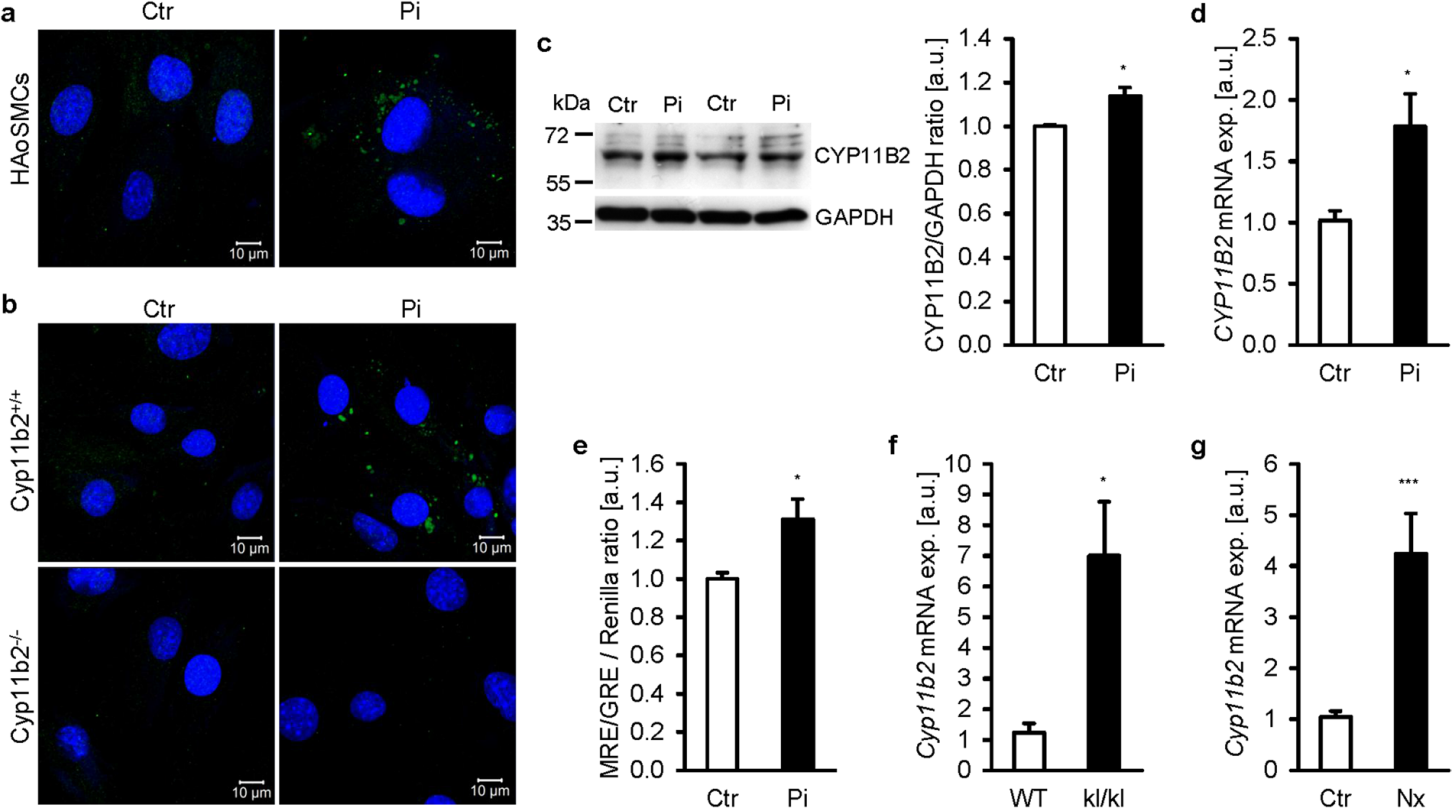
Phosphate induces aldosterone synthase expression in VSMCs. Representative confocal microscopy images showing aldosterone synthase (CYP11B2) protein expression in HAoSMCs (**a**) and in MAoSMCs isolated from aldosterone synthase-deficient mice (Cyp11b2^−/−^) or corresponding wild-type mice (Cyp11b2^+/+^)(**b**) following treatment for 24 hours with (Pi) or without (Ctr) phosphate. Images are representative for four independent experiments. CYP11B2 expression: green labeling, nuclei: blue labeling. Scale bar: 10 μm. (**c**) Representative original Western blots and arithmetic means ± SEM (n = 4; arbitrary units, a.u.) of normalized CYP11B2/GAPDH protein ratio in HAoSMCs following treatment for 24 hours with (Pi) or without (Ctr) phosphate. Arithmetic means ± SEM of *CYP11B2* relative mRNA expression (**d**, n = 6; a.u.) and MRE/GRE-dependent transcriptional activity measured by luciferase reporter assay (**e**, n = 6; a.u.) in HAoSMCs following treatment for 24 hours with (Pi) or without (Ctr) phosphate. Arithmetic means ± SEM (a.u.) of *Cyp11b2* relative mRNA expression in aortic tissue from *kl/kl* mice and corresponding wild-type mice (WT)(**f**, n = 8) and from DBA mice without (CTR) or with subtotal nephrectomy (Nx) (**g**, n = 8). *(p < 0.05) statistically significant vs. control treated HAoSMCs, WT mice or control treated mice, respectively.

To elucidate the underlying mechanisms of MR activation under high phosphate conditions, the expression of vascular CYP11B2 was investigated. *CYP11B2* expression was detectable in HAoSMCs albeit at lower levels than in human adrenocortical carcinoma H295 cells (Supplementary Fig. S3). Cholesterol side-chain cleavage enzyme (*CYP11A1*), steroid 21-hydroxylase (*CYP21A2*), steroidogenic acute regulatory protein (*STARD1*), hydroxy-delta-5-steroid dehydrogenase, 3 beta- and steroid delta-isomerase 1 (*HSD3B1*), hydroxy-delta-5-steroid dehydrogenase, 3 beta- and steroid delta-isomerase 2 (*HSD3B2*) and steroid 11-beta-hydroxylase (*CYP11B1*) were all expressed in HAoSMCs, but at lower levels as compared to H295 cells (Supplementary Figs S3 and S4). Phosphate treatment did not significantly modify *CYP21A2*, *HSD3B2*, *STARD1* and *CYP11B1* mRNA expression, but significantly increased *CYP11A1*, *HSD3B1* and *CYP11B2* mRNA levels in HAoSMCs (Supplementary Fig. S4, Fig. 2d). Furthermore, phosphate treatment did not significantly modify the mRNA expression of renin-angiotensin system components pro-renin receptor (*ATP6AP2*), angiotensin-converting enzyme (*ACE*) and type 1 (*AGTR1*) and type 2 (*AGTR2*) angiotensin II receptors in HAoSMCs (Supplementary Fig. S5).

As shown by confocal microscopy, phosphate treatment stimulated CYP11B2 protein expression in HAoSMCs (Fig. 2a). To confirm antibody specificity in VSMCs, MAoSMCs were isolated from aldosterone synthase-deficient mice (Cyp11b2^−/−^) and corresponding wild-type (Cyp11b2^+/+^) mice. Similarly, phosphate stimulated Cyp11b2 protein expression in Cyp11b2^+/+^ MAoSMCs, but no expression was observed in Cyp11b2^−/−^ MAoSMCs (Fig. 2b). Increased *CYP11B2* mRNA expression was paralleled by elevated CYP11B2 protein abundance in phosphate treated HAoSMCs (Fig. 2c,d). Angiotensin II, a known stimulator of CYP11B2 transcription, significantly increased *CYP11B2* mRNA expression to values similarly high as following phosphate treatment. *CYP11B2* mRNA levels could not be further increased by additional phosphate treatment (Supplementary Fig. S6).

Phosphate treatment did not modify *MR* (*NR3C2*) or glucocorticoid receptor (*GR*, *NR3C1*) expression in HAoSMCs (Supplementary Fig. S7). Most importantly, according to luciferase reporter assay in HAoSMCs, phosphate triggered MR response element/GR response element (MRE/GRE)-dependent transcriptional activity in the absence of exogenous aldosterone (Fig. 2e). *Cyp11b2* mRNA expression was significantly higher in aortic tissue from *kl/kl* mice as compared to corresponding wild-type mice (Fig. 2f) and from mice with subtotal nephrectomy as compared to control treated mice (Fig. 2g). Aortic expression of *Cyp11b2* was further confirmed by qRT-PCR using intron-spanning primers (Supplementary Fig. S8a). However, aldosterone release into the cell culture medium was not detectable by LC-MS/MS, despite a surprisingly increased production of cortisone, dehydroepiandrosterone and testosterone in phosphate treated HAoSMCs (Supplementary Fig. S9).

### Aldosterone synthase-dependent phosphate-induced calcification in VSMCs

**Figure 3:**
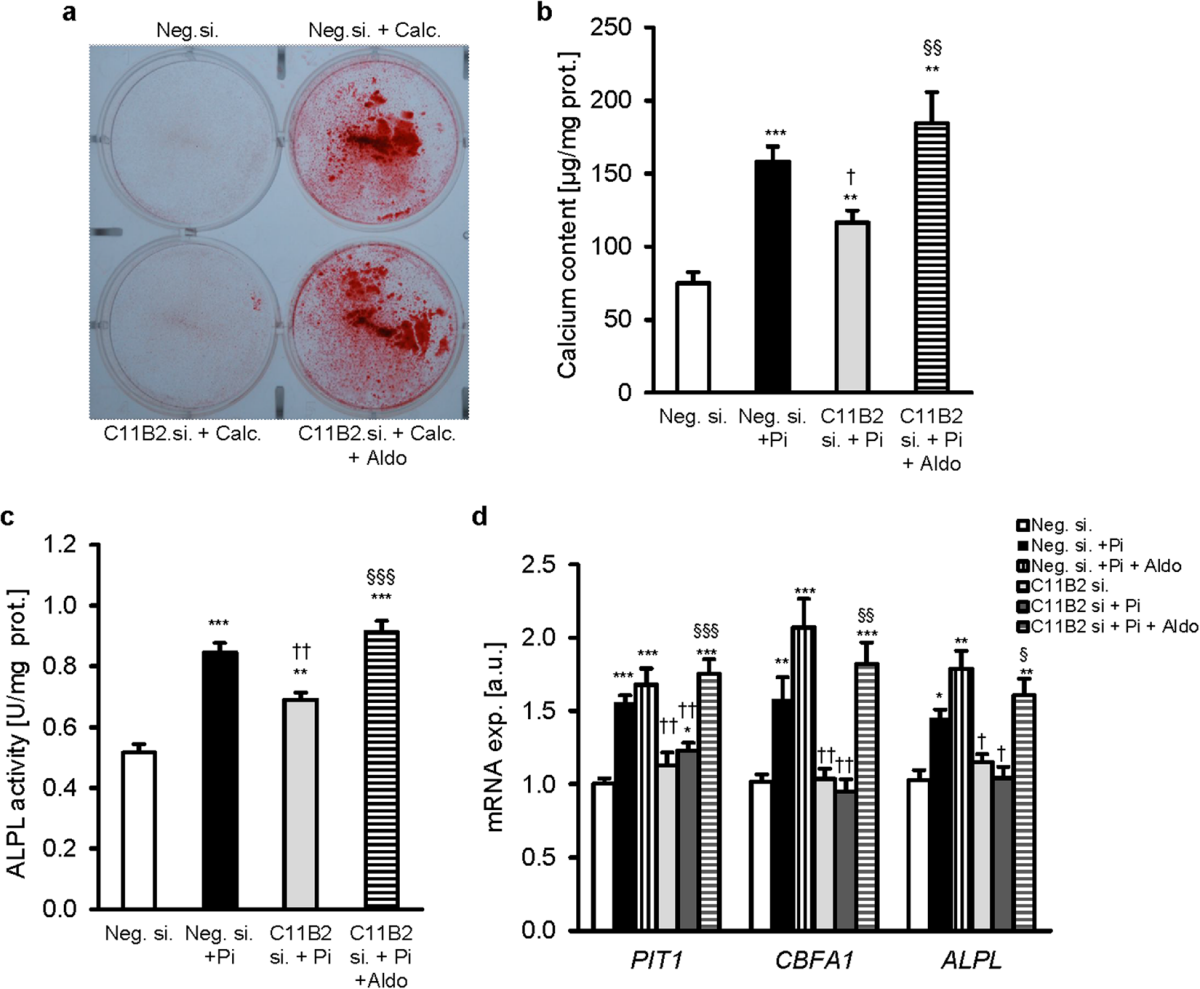
Aldosterone synthase inhibition reduces phosphate-induced vascular calcification *in vitro*. (**a**) Representative original images showing Alizarin red staining in HAoSMCs silenced with negative control siRNA (Neg.si.) or CYP11B2 siRNA (C11B2si.) and treated with or without calcification medium and with or without additional treatment with 100 nM aldosterone (Aldo). Images are representative for four independent experiments. The calcified areas are shown as red staining. Arithmetic means ± SEM of calcium content (**b**, n = 12; µg/mg protein), alkaline phosphatase activity (**c**, n = 8; units/mg protein) and of *PIT1*, *CBFA1* and *ALPL* relative mRNA expression (**d**, n = 14, arbitrary units, a.u.) in HAoSMCs silenced with Neg.si. or C11B2si. and treated with (Pi) or without (Ctr) phosphate and with or without additional treatment with 100 nM aldosterone (Aldo). *(p < 0.05), **(p < 0.01), ***(p < 0.001) statistically significant vs. Neg.si. silenced HAoSMCs. ^†^(p < 0.05), ^††^(p < 0.01) statistically significant vs. Pi treated Neg.si. silenced HAoSMCs. ^§^(p < 0.05), ^§§^(p < 0.01), ^§§§^(p < 0.001) statistically significant between Pi and Pi + Aldo treated C11B2si silenced HAoSMCs.

Further experiments were performed to investigate, whether the increased expression of CYP11B2 contributes to osteo-/chondrogenic reprogramming of HAoSMCs following phosphate treatment. As a result, silencing of the CYP11B2 gene in HAoSMCs (Supplementary Fig. S10a,b) blunted the phosphate-induced MRE/GRE-dependent transcriptional activity as compared to negative control silenced HAoSMCs (Supplementary Fig. S10c). The mRNA expression of the smooth muscle cell specific marker α-smooth muscle actin (*ACTA2)* was significantly decreased by phosphate treatment in negative control silenced HAoSMCs, effects ameliorated following silencing of CYP11B2 gene (Supplementary Fig. S11a). Phosphate treatment significantly increased calcium deposition (Fig. 3a,b), alkaline phosphatase activity (Fig. 3c) and *PiT1*, *CBFA1* and *ALPL* mRNA expression (Fig. 3d) in negative control silenced HAoSMCs, effects ameliorated in HAoSMCs silenced with CYP11B2 siRNA. The inhibitory effects of CYP11B2 silencing on phosphate-induced osteo-/chondrogenic transformation of HAoSMCs were abolished by addition of exogenous aldosterone. Without phosphate or aldosterone treatment, silencing of CYP11B2 did not significantly modify the transcript levels of osteo-/chondrogenic markers in HAoSMCs (Fig. 3d).

**Figure 4:**
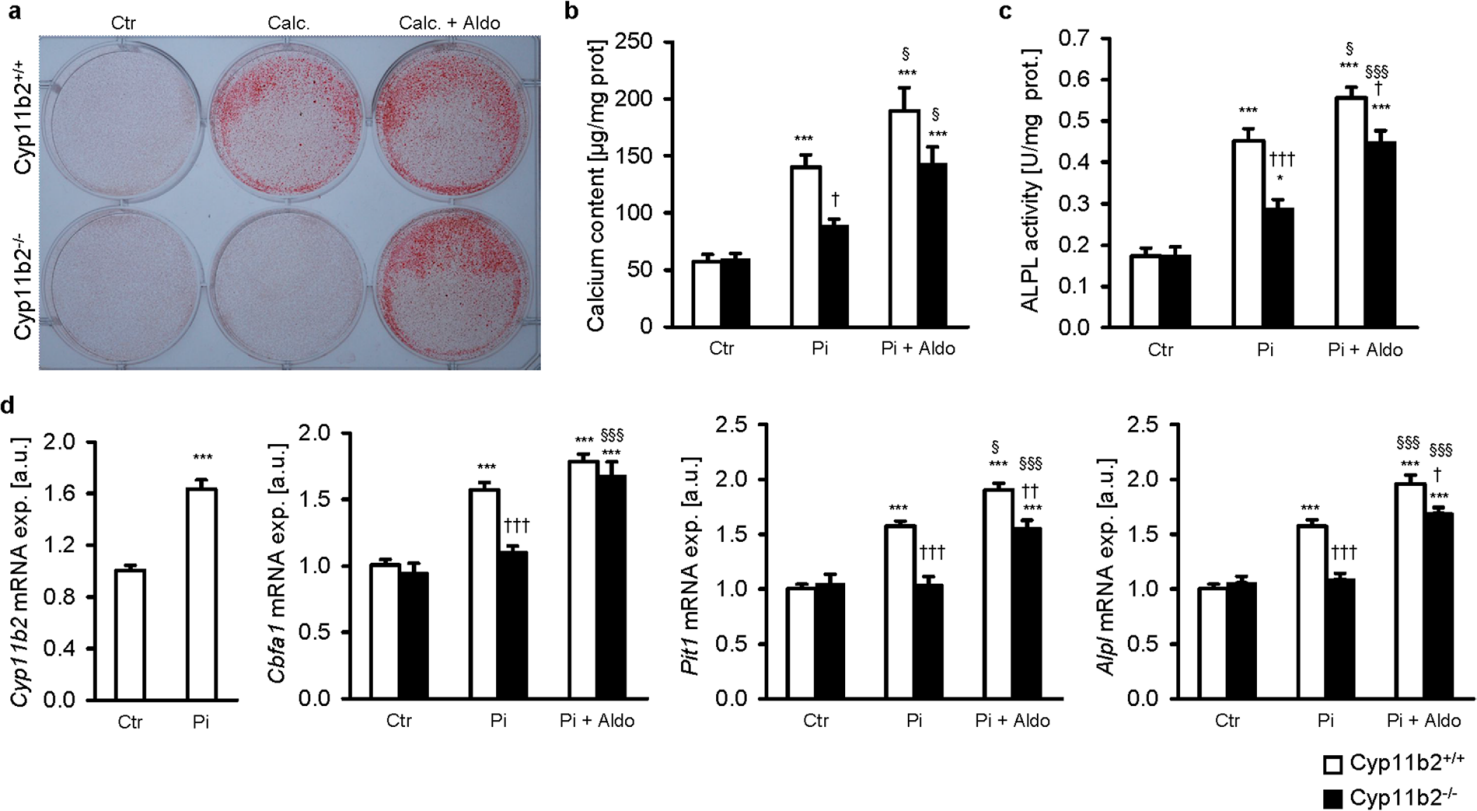
Aldosterone synthase deficiency reduces phosphate-induced vascular calcification *in vitro*. (**a**) Representative original images showing Alizarin red staining in MAoSMCs isolated from aldosterone synthase-deficient mice (Cyp11b2^−/−^) or corresponding wild-type mice (Cyp11b2^+/+^) treated with or without calcification medium and with or without additional treatment with 100 nM aldosterone (Aldo). Images are representative for three independent experiments. The calcified areas are shown as red staining. Arithmetic means ± SEM of calcium content (**b**, n = 9; µg/mg protein), alkaline phosphatase activity (**c**, n = 8; units/mg protein) and of *Cyp11b2*, *Pit1*, *Cbfa1* and *Alpl* relative mRNA expression (**d**, n = 9, a.u.) in MAoSMCs isolated from Cyp11b2^−/−^ or Cyp11b2^+/+^ mice treated with (Pi) or without (Ctr) phosphate and with or without additional treatment with 100 nM aldosterone (Aldo). *(p < 0.05), ***(p < 0.001) statistically significant vs. respective control treated MAoSMCs. ^†^(p < 0.05), ^††^(p < 0.01) ^†††^(p < 0.001) statistically significant vs. respective wild-type MAoSMCs. ^§§^(p < 0.01), ^§§§^(p < 0.001) statistically significant vs. respective Pi treated MAoSMCs.

Similar observations were made when treating MAoSMCs isolated from Cyp11b2^−/−^ and Cyp11b2^+/+^ mice with phosphate. In control treated MAoSMCs, no significant difference on the smooth muscle cell specific marker *Acta2* mRNA expression was noted between the genotypes. *Acta2* mRNA expression was significantly decreased by phosphate treatment in Cyp11b2^+/+^ MAoSMCs (Supplementary Fig. S11b). Phosphate treatment increased the calcium deposition (Fig. 4a,b), alkaline phosphatase activity (Fig. 4c) and the mRNA expression of *Cyp11b2*, *Pit1*, *Cbfa1* and *Alpl* in Cyp11b2^+/+^ MAoSMCs (Fig. 4d, Supplementary Fig. S8b). These effects were abrogated in the Cyp11b2^−/−^ MAoSMCs. Addition of exogenous aldosterone blunted the effects of Cyp11b2 deficiency during phosphate treatment (Fig. 4). However, Cyp11b2^−/−^ MAoSMCs still displayed lower alkaline phosphatase activity, *Pit1* and *Alpl* mRNA expression as compared to Cyp11b2^+/+^ MAoSMCs during treatment with phosphate and exogenous aldosterone.

### Role of *APEX1*, a transcriptional repressor of *CYP11B2* in phosphate-induced VSMC calcification

**Figure 5:**
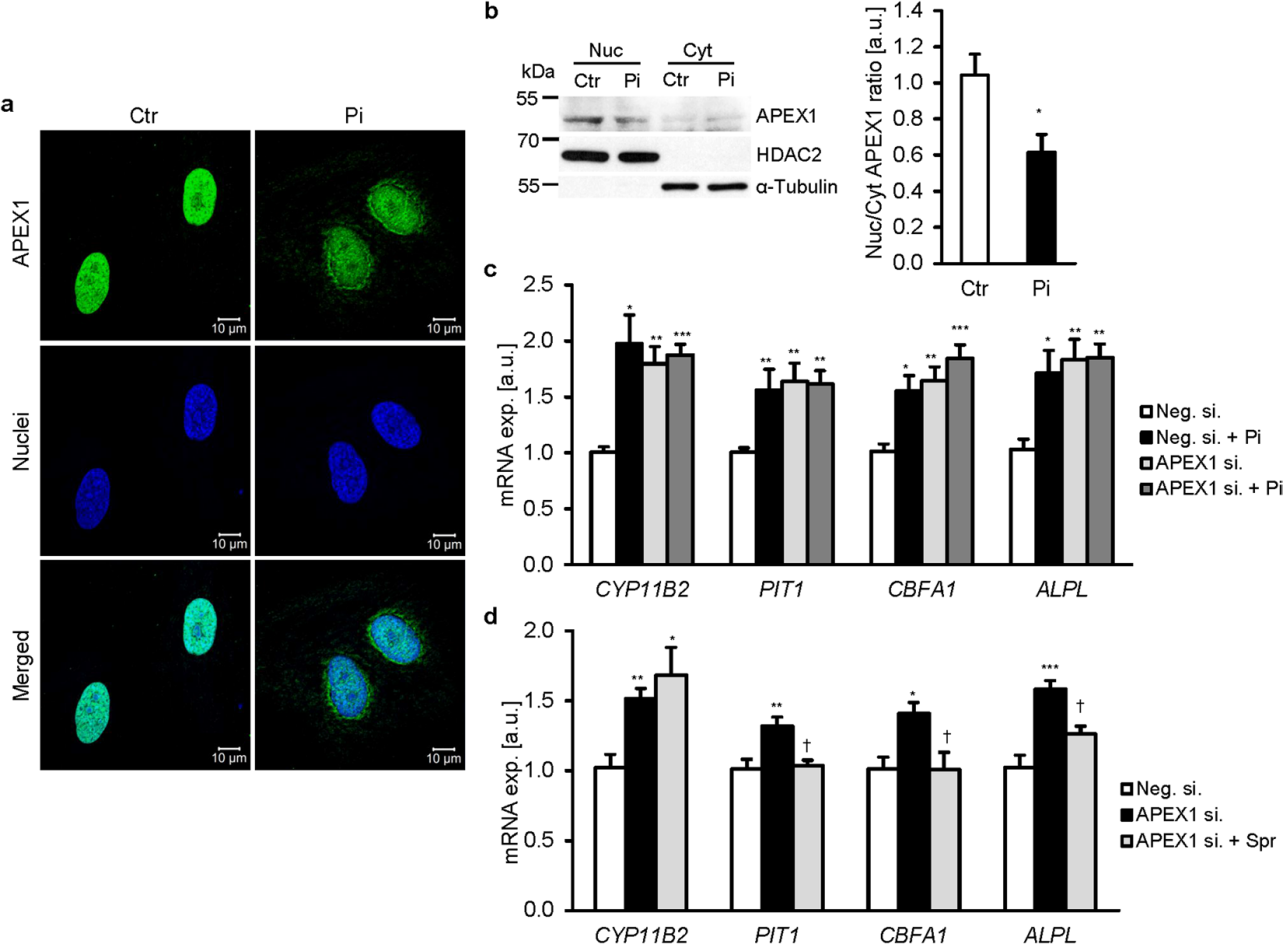
APEX1 represses aldosterone synthase expression in HAoSMCs. (**a**) Representative confocal microscopy images of APEX1 protein expression in HAoSMCs following treatment for 24 hours with (Pi) or without (Ctr) phosphate. Images are representative for four independent experiments. APEX1 expression: green labeling, nuclei: blue labeling. Scale bar: 10 μm. (**b**) Representative original Western blots and arithmetic means ± SEM (n = 5; arbitrary units, a.u.) of normalized nuclear to cytoplasmic APEX1 protein ratio normalized to HDAC2 and α-Tubulin protein in the nuclear fraction and cytoplasmic fraction respectively, in HAoSMCs following treatment for 24 hours with (Pi) or without (Ctr) phosphate. *(p < 0.05) statistically significant vs. control treated HAoSMCs. Arithmetic means ± SEM (a.u.) of *CYP11B2*, *PIT1*, *CBFA1* and *ALPL* relative mRNA expression in HAoSMCs silenced for 48 hours with negative control siRNA (Neg.si.) or APEX1 siRNA (APEX1si.) and treated for 24 hours with (Pi) or without (Ctr) phosphate (**c**, n = 8) or with or without 10 µM spironolactone (Spr) (**d**, n = 6). *(p < 0.05), **(p < 0.01), ***(p < 0.001) statistically significant vs. Neg.si. silenced HAoSMCs. ^†^(p < 0.05) statistically significant vs. APEX1si. silenced HAoSMCs.

To explore the mechanisms underlying the up-regulation of CYP11B2 by phosphate, the role of APEX1 (apurinic-apyrimidinic endonuclease 1), a transcriptional repressor of the CYP11B2 gene^41^, was investigated. As illustrated in Fig. 5a,b, phosphate treatment caused the nuclear export of APEX1 in HAoSMCs. *APEX1* mRNA and protein expression were not significantly modified by phosphate (Supplementary Fig. S12a,b).

The functional relevance of APEX1 was further investigated by silencing of the APEX1 gene in HAoSMCs (Supplementary Fig. S12c). Silencing of APEX1 in HAoSMCs was followed by a significant increase of *CYP11B2*, *PIT1*, *CBFA1* and *ALPL* mRNA levels to values similarly high as following treatment with phosphate (Fig. 5c). No additive effects of phosphate treatment and APEX1 silencing were observed. The increase of *CYP11B2* expression following APEX1 silencing was not modified by additional treatment with spironolactone (Fig. 5d). In contrast, addition of spironolactone abrogated the increase of *PIT1*, *CBFA1* and *ALPL* mRNA levels following APEX1 silencing, suggesting that APEX1 silencing caused spironolactone-sensitive osteogenic transformation via up-regulation of CYP11B2. ATF2, another transcriptional regulator of the CYP11B2 gene was apparently not involved in the regulation of *CYP11B2* expression by phosphate, as ATF2 silencing did not prevent the phosphate-induced *CYP11B2* mRNA expression (Supplementary Fig. S13).

**Figure 6:**
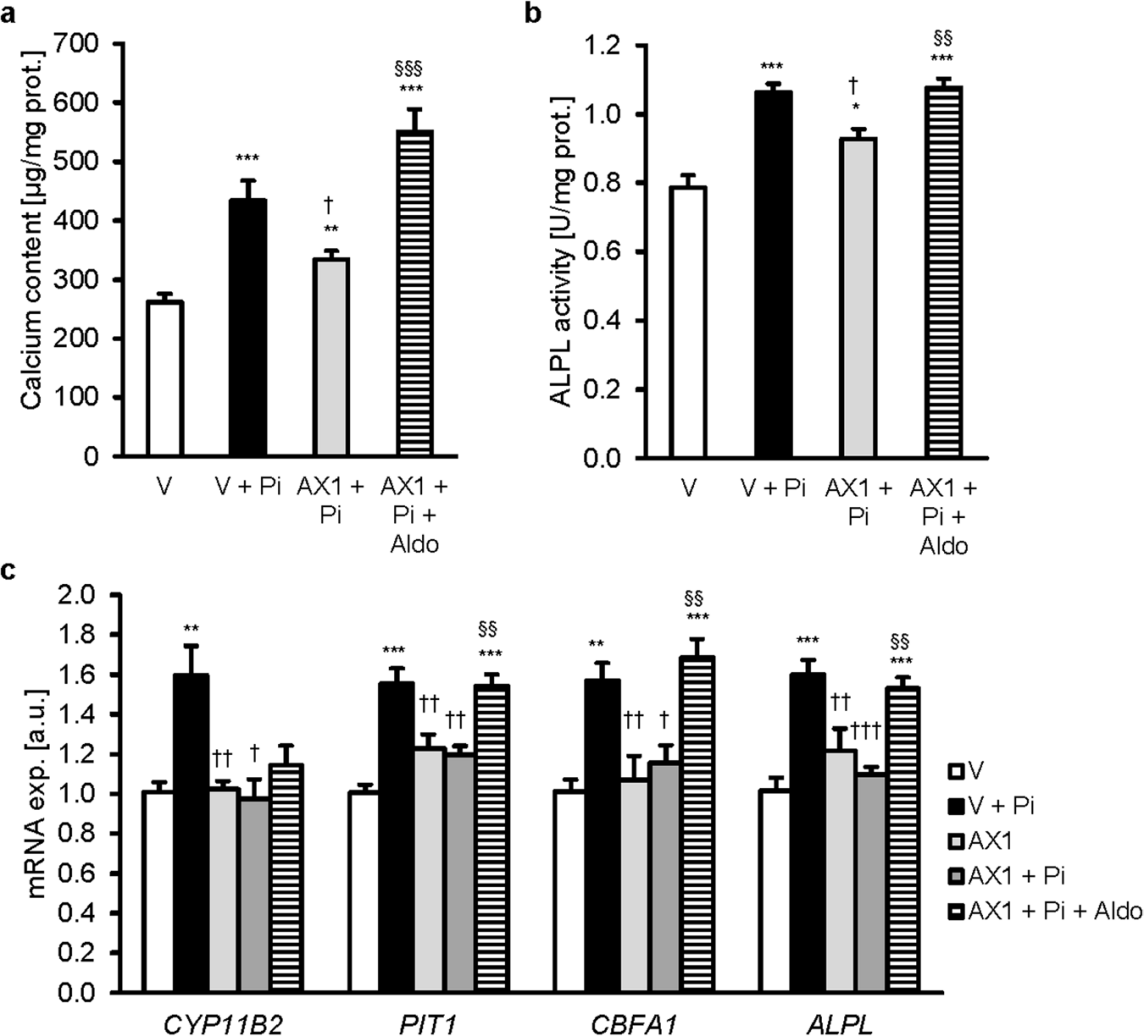
APEX1 overexpression ameliorates phosphate-induced vascular calcification *in vitro*. Arithmetic means ± SEM of calcium content (**a**, n = 8; µg/mg protein), alkaline phosphatase activity (**b**, n = 8; units/mg protein) and of *CYP11B2*, *PIT1*, *CBFA1* and *ALPL* relative mRNA expression (**c**, n = 8; a.u.) in HAoSMCs transfected with empty vector (V) or APEX1 (AX1) and treated with (Pi) or without (Ctr) phosphate and with or without additional treatment with 100 nM aldosterone (Aldo). *(p < 0.05), **(p < 0.01), ***(p < 0.001) statistically significant vs vector transfected HAoSMCs. ^†^(p < 0.05) statistically significant vs Pi treated vector transfected HAoSMCs. ^§§^(p < 0.01), ^§§§^(p < 0.001) statistically significant between Pi and Pi + Aldo treated APEX1 transfected HAoSMCs.

Additional experiments tested whether overexpression of human APEX1 negatively regulates osteo-/chondrogenic transformation of HAoSMCs (Supplementary Fig. S14a). As compared to empty vector transfected HAoSMCs, transfection of HAoSMCs with APEX1 significantly ameliorated phosphate-induced increase of calcium deposition (Fig. 6a) and alkaline phosphatase activity (Fig. 6b) and blunted the increase of *CYP11B2* mRNA expression (Fig. 6c). APEX1 overexpression did not modify the calcium content or ALPL activity under control conditions (Supplementary Fig. S14b,c). The phosphate-induced mRNA expression of *PIT1*, *CBFA1* and *ALPL* was blunted by APEX1 overexpression (Fig. 6c). Addition of exogenous aldosterone did not significantly modify *CYP11B2* mRNA expression, but abrogated the effects of APEX1 overexpression on *PIT1*, *CBFA1* and *ALPL* mRNA levels.

### Effect of spironolactone and adrenalectomy on vascular osteoinductive transformation in klotho-hypomorphic mice

In order to estimate the *in vivo* significance of vascular Cyp11b2 in the early phase of vascular osteogenic transformation, experiments were performed in *kl/kl* mice and corresponding wild-type mice rescued by pretreatment with NH_4_Cl (0.28 M) added to the drinking water^42^. Following discontinuation of NH_4_Cl, mice were treated with either control drinking water, spironolactone in drinking water (80 mg/l) or adrenalectomy for 5 weeks. The NH_4_Cl pre-treatment abolished the strong phenotype of growth retardation of *kl/kl* mice and thereby allowed for adrenalectomy treatment (Supplementary Table S1). Plasma calcium and phosphate concentrations were increased in *kl/kl* mice irrespective of the treatments. Blood pressure was similar in all groups. As shown in Supplementary Fig. S15a, aortic *Cyp11b2* mRNA levels were significantly higher in control drinking water treated *kl/kl* mice than in wild-type mice, but were not significantly modified by spironolactone treatment or adrenalectomy. Aortic *Pit1*, *Cbfa1, Msx2*, and *Alpl* mRNA levels were all significantly higher in control drinking water treated *kl/kl* mice than in wild-type mice and were significantly decreased by spironolactone treatment, but not significantly modified by adrenalectomy (Supplementary Fig. S15b). Similar alterations were observed on Cyp11b2, Cbfa1 and Msx2 protein abundance (Supplementary Fig. S15c).

**Figure 7:**
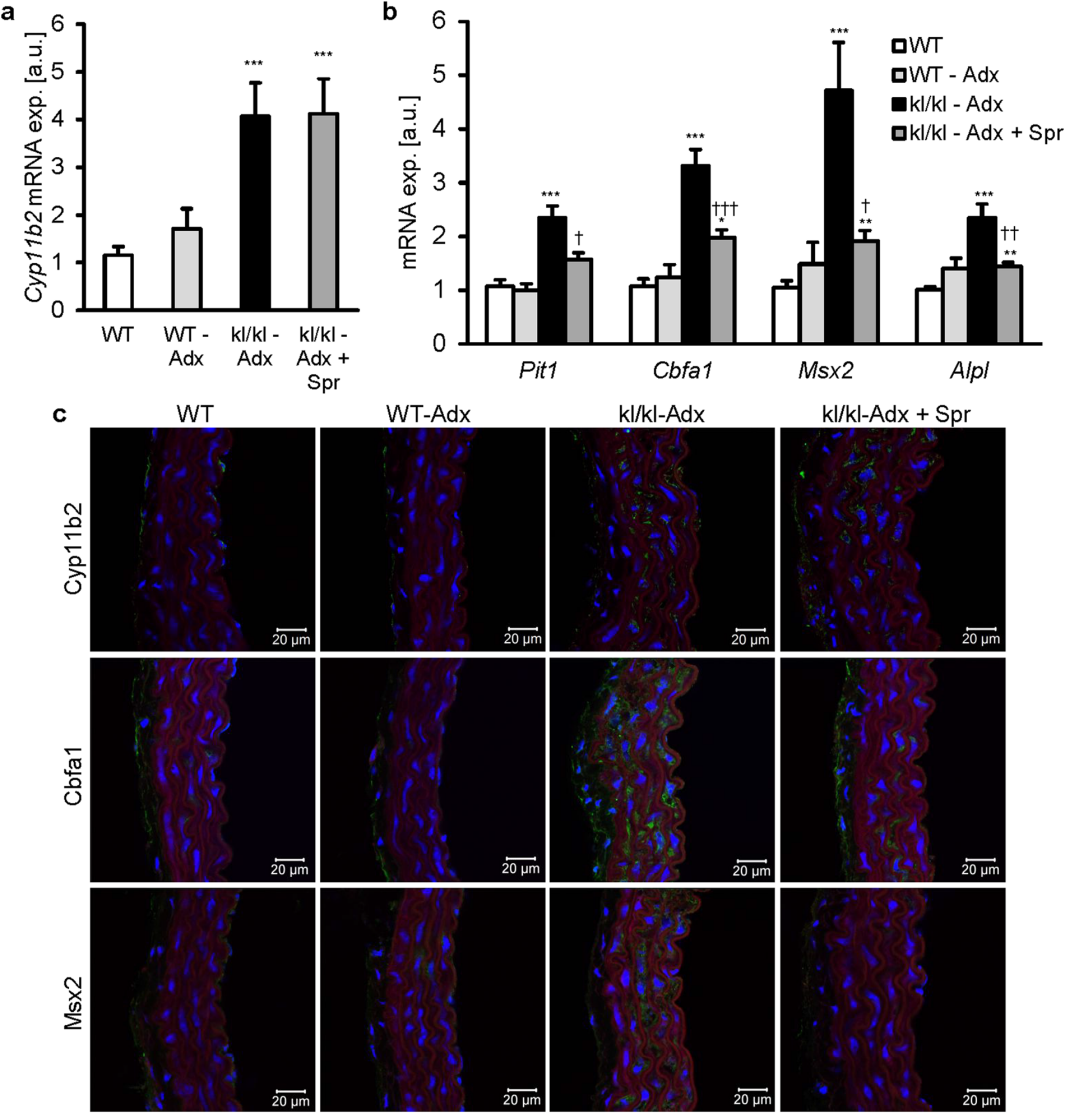
Vascular osteoinductive transformation in adrenalectomized *kl/kl* mice is ameliorated by spironolactone. Arithmetic means ± SEM (n = 10–11; arbitrary units, a.u) of aortic *Cyp11b2* (**a**) and *Pit1*, *Cbfa1, Msx2* and *Alpl* (**b**) relative mRNA expression in wild-type (WT) and *kl/kl* mice following discontinuation of dietary rescue and treatment without or with spironolactone (Spr) and adrenalectomy (Adx) for 5 weeks. *(p < 0.05), **(p < 0.01), ***(p < 0.001) statistically significant vs. WT control mice. ^†^(p < 0.05), ^††^(p < 0.01) statistically significant between *kl/kl-*Adx and *kl/kl*-Adx + Spr. (**c**) Representative confocal microscopy images showing Cyp11b2, Cbfa1 and Msx2 protein expression in aortic tissues from wild-type (WT) and *kl/kl* mice following discontinuation of dietary rescue and treatment without or with spironolactone (Spr) and adrenalectomy (Adx) for 5 weeks. Images are representative of four mice per group. Protein expression: green labeling; nuclei: blue labeling and actin staining: red labeling. Scale bar: 20 µm.

A further series of experiments explored whether spironolactone influences aortic osteo-/chondrogenic transformation in adrenalectomized mice (Supplementary Table S2). As illustrated in Fig. 7a, aortic *Cyp11b2* transcript levels were significantly higher in adrenalectomized *kl/kl* mice than in wild-type mice irrespective of additional spironolactone treatment. The mRNA levels of *Pit1*, *Cbfa1, Msx2* and *Alpl* were all higher in adrenalectomized *kl/kl* mice than in wild-type mice, but significantly decreased following spironolactone treatment of adrenalectomized *kl/kl* mice (Fig. 7b). Again, the effects on transcript levels were paralleled by the respective alterations of Cyp11b2, Cbfa1 and Msx2 protein expression (Fig. 7c).

### Aldosterone synthase expression in human coronary arteries

**Figure 8:**
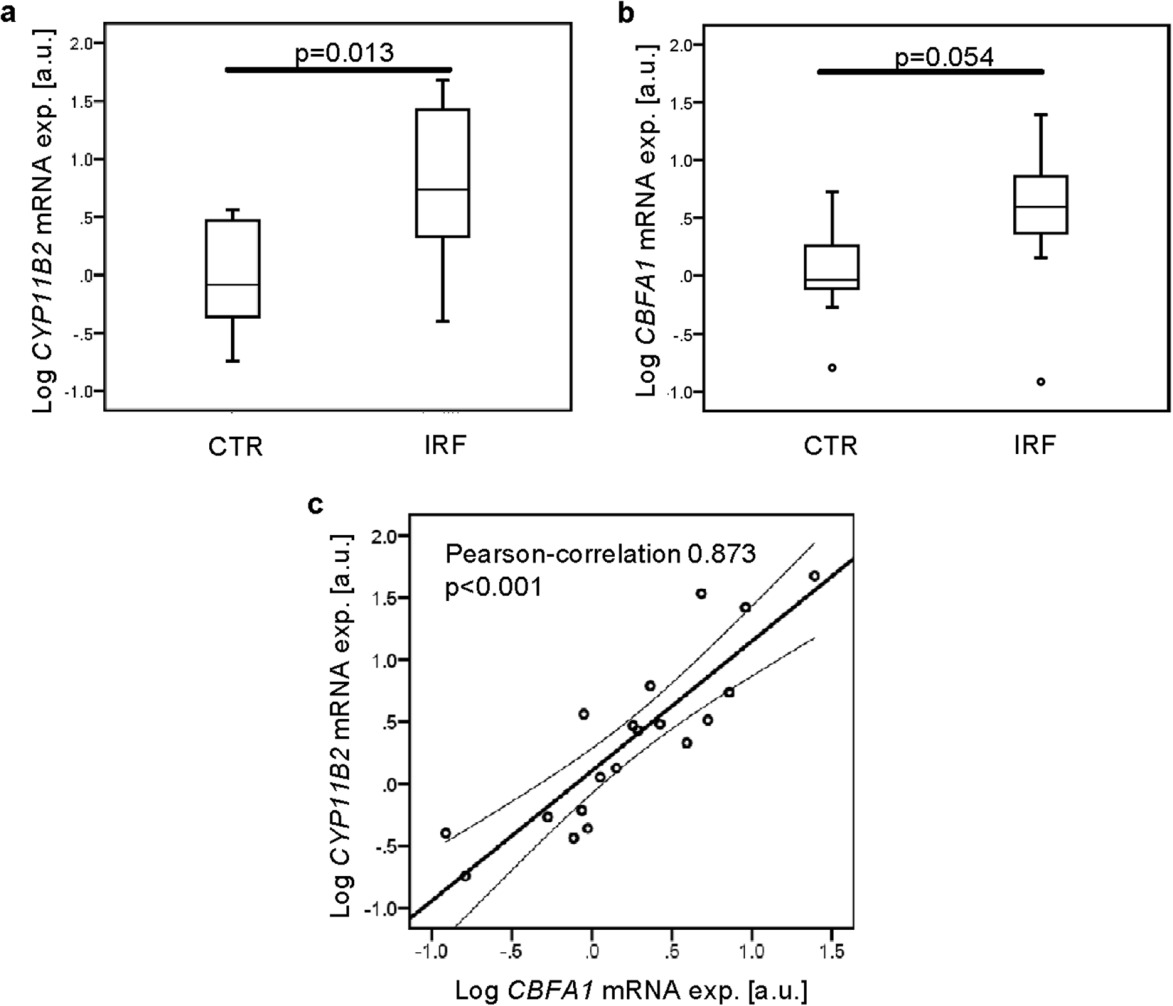
Aldosterone synthase and *CBFA1* expression in human coronary arteries. Boxplots of log-transformed *CYP11B2* (**a**, arbitrary units, a.u.) and *CBFA1* (**b**, a.u.) relative mRNA expression in right coronary artery tissue from patients with maintained (CTR, n = 10) and impaired (IRF, n = 9) renal function. (**c**) Association of log-transformed *CYP11B2* (a.u.) and *CBFA1* (a.u.) relative mRNA expression in right coronary artery tissue from patients with maintained (n = 10) and impaired (n = 9) renal function. P values are indicated in the figure.

In order to test whether CYP11B2 is expressed in human arteries, the expression of *CYP11B2* was quantified in right coronary arteries from patients with impaired (IRF, n = 9) or maintained renal function (CTR, n = 10, Supplementary Table S3). Right coronary artery biopsies were isolated and the mRNA expression of *CYP11B2* and *CBFA1* was determined. In coronary arteries from patients with impaired renal function, log-transformed *CYP11B2* mRNA expression was significantly increased as compared to patients with maintained renal function (Fig. 8a). Log-transformed *CBFA1* mRNA expression tended to be higher in vessels from patients with impaired renal function than in patients with maintained renal function, a difference, however, not reaching statistical significance (p = 0.054, Fig. 8b). *CBFA1* expression was not significantly correlated with plasma aldosterone levels (Pearson correlation coefficient: −0.007, p = 0.978; n = 17). However, in both groups, the arterial *CYP11B2* mRNA expression showed a significant correlation with *CBFA1* mRNA expression (Fig. 8c).

## Discussion

The present observations suggest a novel role of vascular CYP11B2. CYP11B2 expression was up-regulated by elevated extracellular phosphate concentrations and CYP11B2 was required for the triggering of osteo-/chondrogenic reprogramming of VSMCs following phosphate treatment. Vascular CYP11B2 was similarly up-regulated in hyperphosphatemic *kl/kl* mice, and in the subtotal nephrectomy renal failure mouse model. Vascular CYP11B2 was further increased in patients with early stages of renal failure, but the results in brain dead multi organ donors should be interpreted with caution. Together, these findings suggest that hyperphosphatemia increases vascular CYP11B2 transcription, which is a prerequisite for VSMC osteo-/chondrogenic transformation.

Expression of functional CYP11B2 in the vasculature has been reported earlier^33, 43^. However, the significance of vascular aldosterone production remained controversial^38^. As observed in this study, VSMCs express enzymes involved in the generation of aldosterone precursors from cholesterol and therefore may be able to produce aldosterone *de novo*. Human pulmonary artery endothelial cells express CYP11B2 and were described to synthesize aldosterone during hypoxia^33^. In these cells, hypoxia did not affect CYP11B2 expression, but increased vascular aldosterone synthesis by up-regulation of StAR (encoded by STARD1 gene) expression, which facilitates the first and rate-limiting step in aldosterone biosynthesis^33^. In VSMCs, phosphate treatment did not affect StAR expression, but up-regulated CYP11B2, suggesting that hypoxia and phosphate exposure act via different mechanisms on the vascular aldosterone producing system. Phosphate treatment increased production and release of glucocorticoids and androgens by VSMCs, but aldosterone could not be detected. Aldosterone secreted by VSMCs may be at low levels not detectable by the employed method or remain and act intracellularly. Low aldosterone levels within the VSMCs may be sufficient for MR activation. Furthermore, vascular CYP11B2 may rather produce 18-hydroxycorticosterone, which was not included in our protocol. A profound effect of vascular CYP11B2 on aldosterone levels may also require precursors from the circulation. Nevertheless, CYP11B2 silencing blunted phosphate-induced MR-dependent transcriptional activity, suggesting that MR activation is, at least partly, dependent on CYP11B2 up-regulation. In accordance, functional effects of CYP11B2 in the vasculature have been reported^44, 45^ and appear important especially during pathological conditions.

A potential significance of vascular auto/paracrine MR activation is underscored by the inhibitory effect of the MR blocker spironolactone on aortic osteo-/chondrogenic transformation in adrenalectomized *kl/kl* mice. At least in those mice, local aldosterone synthesis rather than adrenal aldosterone release appears to be instrumental for VSMCs osteoinduction. MR-independent effects of spironolactone cannot be ruled out^46^, but the effects of spironolactone and eplerenone were mimicked by MR silencing *in vitro*, an observation confirming a crucial role for MR activation in vascular calcification. However, a ligand-independent transactivation or glucocorticoid-dependent activation of the MR could contribute to the observed effects^47^. Glucocorticoids are able to activate MR and promote vascular calcification^47^. The effects of androgens on vascular calcification are still controversial. Androgens may induce vascular calcification through androgen receptor (AR) activation^48^, but may also have anti-calcifying effects by AR-dependent up-regulation of Gas6 transcription^49^.

Up-regulation of vascular CYP11B2 has also been demonstrated in vascular tissue of diabetic rats^34^. Vasculo-protective properties have further been associated with spironolactone in diabetes models^50^. It is therefore tempting to speculate, that vascular CYP11B2 could also contribute to osteoinductive remodeling of vascular tissue during diabetes.

The present observations further shed light on the mechanism underlying the effect of extracellular phosphate on increased CYP11B2 expression. Elevated phosphate triggered the nuclear export of the transcriptional repressor APEX1, which has been shown to repress *CYP11B2* mRNA expression^41^. The transcription factor ATF2, a positive regulator of CYP11B2 gene expression was apparently not involved in the up-regulation of CYP11B2 by phosphate^41^. APEX1 silencing up-regulated *CYP11B2* mRNA expression and mimicked the effects of phosphate in HAoSMCs. Conversely, APEX1 overexpression ameliorated the phosphate-induced HAoSMCs osteo-/chondrogenic transformation. APEX1 is thus a key factor in VSMCs *CYP11B2* expression. Nuclear export of APEX1 has previously been shown following nitrosative stress, which was also suggested to participate in the regulation of vascular calcification^51, 52^. APEX1 exported from the nucleus is further triggered by the histone deacetylase inhibitor trichostatin-A^53^, which promotes VSMC calcification via up-regulation of alkaline phosphatase^54^.

VSMCs have previously been shown to express the MR^55^ and MR activation has been shown to augment vascular calcification via direct up-regulation of PiT1 gene expression^15–17^. In view of the present and previous observations, the following sequence of events may lead to vascular calcification: Hyperphosphatemia leads to down-regulation of nuclear APEX1, which is in turn followed by up-regulation of CYP11B2, MR activation, MR-dependent up-regulation of PIT1 and subsequent triggering of osteogenic transformation. According to the present observations, the protective effects of MR blockade on osteoinductive signaling in VSMCs may be, at least in part, independent of elevated circulating aldosterone levels.

First clinical trials indicate that spironolactone treatment reduces morbidity and mortality in hemodialysis patients^24^. MR inhibition may lead to transient reduction of renal function and hyperkalemia, but is in general well tolerated in CKD patients^24, 56^. In CKD patients, elevated aldosterone levels were observed, increasing with advanced renal insufficiency^23^. Vascular CYP11B2 expression is triggered by an increase of extracellular phosphate concentration via an APEX1-dependent mechanism and is decisive for the triggering of osteo-/chondrogenic reprogramming of VSMCs during elevated phosphate conditions. The present observations therefore advocate the therapeutic use of MR blockade in conditions with hyperphosphatemia such as CKD even in the absence of hyperaldosteronism.

## Methods

### Cell culture of VSMCs

Primary human aortic smooth muscle cells (HAoSMCs; Thermo Fisher Scientific) were cultured in Waymouth’s MB 752/1 medium and Ham’s F-12 nutrient mixture (1:1, Thermo Fisher Scientific) supplemented with 10% FBS (Thermo Fisher Scientific) and 100 U/ml penicillin and 100 µg/ml streptomycin (Thermo Fisher Scientific). HAoSMCs were grown to confluency and used from passages 4 to 11 (n indicates number of independent experiments performed at different passages of the cells). At least 6 different batches of HAoSMCs were used during the course of this study and each experiment was performed in at least 2 different batches of HAoSMCs depending on the availability of the cells.

Primary mouse aortic smooth muscle cells (MAoSMCs) were isolated from aldosterone synthase-deficient or corresponding wild-type mice^57^. VSMCs were obtained from pooled aortic tissue by a modification of the protocol described by Owens *et al*.^58^. Animals were sacrificed and the thoracic aorta from below the arch to the proximity of the diaphragm was removed and placed in Hanks’ balanced salt solution (HBSS, Thermo Fischer Scientific). The fat and fibrous tissue around the aorta was removed. The aortic tissue was then incubated for 10 minutes at 37 °C, 5% CO_2_ in HBSS enzyme solution containing 125 U/ml collagenase type II (Worthington Biochemical), 0.744 U/ml elastase (Sigma Aldrich), 1 mg/ml soybean trypsin inhibitor (Sigma Aldrich), 100 U/ml penicillin and 100 µg/ml streptomycin. The adventitia was removed and the luminal surface scraped with forceps to remove endothelial cells. The aortic tissue was then cut into 1 mm pieces and incubated in HBSS enzyme solution for the final digestion for another 2 hours at 37 °C, 5% CO_2_ with trituration every 30 minutes. The resulting cell suspension was centrifuged at 1200 rpm for 1 minute and washed with DMEM/F12 medium (Thermo Fisher Scientific) supplemented with 20% FBS and 100 U/ml penicillin and 100 µg/ml streptomycin. The cells were suspended in cell culture medium and transferred into 48-well plates leaving them undisturbed for 7 days. The media was then replaced every 2–3 days. From passage 2, MAoSMCs were grown to confluence in DMEM/F12 medium supplemented with 10% FBS and 100 U/ml penicillin and 100 µg/ml streptomycin and used for experiments from passages 3 to 5. N indicates the number of independent experiments performed at different passages of the cells from three independent MAoSMCs preparations.

The media was changed to 10% charcoal-stripped FBS media (Sigma Aldrich) 24 hours prior to each experiment to reduce the effects of endogenous ligands. For silencing experiments, HAoSMCs were transfected with 10 nM MR siRNA (ID no. s8839), 10 nM CYP11B2 siRNA (ID no. s3869), 10 nM APEX1 siRNA (ID no. s1445), 10 nM ATF2 siRNA (ID no. s3493) or 10 nM negative control siRNA (ID no. 4390843)(Ambion, Thermo Fisher Scientific) using the siPORT amine transfection agent (Ambion, Thermo Fisher Scientific) according to the manufacturer’s protocol. HAoSMCs were transfected with 2 µg DNA encoding human APEX1 (Source BioScience LifeSciences) in pCMV-SPORT6 or with empty pCMV-SPORT6 vector as control using X-tremeGENE HP DNA transfection reagent (Roche Applied Science) according to the manufacturer’s protocol. Silencing and transfection efficiency were verified by qRT-PCR. Unless indicated otherwise, VSMCs were treated for 24 hours (for qRT-PCR, WB, Luciferase assay) or 7 days (for ALPL activity assay) with 2 mM β-glycerophosphate (Sigma-Aldrich) and with 100 nM aldosterone, 100 nM angiotensin II, 10 µM spironolactone or 10 µM eplerenone (stock dissolved in DMSO, Sigma-Aldrich). Equal amounts of vehicle were used as control. Treatment for 14 days with 3 mM sodium phosphate buffer (Sigma-Aldrich) or for 10 days with 10 mM β-glycerophosphate and 1.5 mM CaCl_2_ (Sigma-Aldrich) were used as calcification media for the calcium content measurements or Alizarin Red staining, respectively^59^. Fresh media with agents were added every 2–3 days.

### Culture of H295 cells

Human adrenocortical carcinoma H295 cells (kindly provided by Prof. Dr. F. Beuschlein, Medizinische Klinik und Poliklinik IV, Munich, Germany) were routinely cultured in DMEM/F12 medium (Thermo Fisher Scientific) supplemented with 1% ITS + premix (BD Biosciences), 2.5% Nu-serum (BD Biosciences) and 100 U/ml penicillin and 100 µg/ml streptomycin (Thermo Fisher Scientific).

### Animal experiments

All animal experiments were conducted according to the guidelines of the American Physiological Society as well as the German law for the welfare of animals and were approved by local authorities (Regierungspräsidium Tübingen). The origin and background of aldosterone synthase-deficient mice and of klotho-hypomorphic (*kl/kl*) mice was described previously^57, 60, 61^. *Kl/kl* and corresponding wild-type (WT) mice without rescue diet treatment were sacrificed and aortic tissue snap frozen in liquid nitrogen. To allow for surgical intervention, *kl/kl* mice and WT mice were fed with NH_4_Cl (0.28 M, Sigma-Aldrich) added to the drinking water for phenotypical rescue^42^. The drinking solution was discontinued and male mice were randomized into treatment groups of control drinking water or spironolactone containing drinking water (80 mg/l, Sigma-Aldrich) at the age of 5–6 weeks. Where indicated, bilateral adrenalectomy was surgically performed under isoflurane anaesthesia. Analgesia was performed by subcutaneous injection of buprenorphin (0.05 mg/kg BW). All adrenalectomized mice received 0.9% NaCl in either control drinking water or spironolactone containing drinking water. Blood pressure was assessed from tail cuff measurements before the end of the observational period. After 5 weeks of treatment, animals were sacrificed, blood was obtained by retro-orbital puncture and murine tissues snap frozen in liquid nitrogen or fixed in 4% paraformaldehyde. The plasma phosphate and calcium concentrations were determined utilizing a photometric method (FUJI FDC 3500i, Sysmex). The subtotal nephrectomy in mice was described previously^62^.

### Calcium content

VSMCs were decalcified for 24 hours at 4 °C in 0.6 M HCl. Calcium content was determined colorimetrically using QuantiChrom Calcium assay kit (BioAssay Systems) according to the manufacturer’s protocol. VSMCs were lysed with 0.1 M NaOH/0.1% SDS. Calcium content was normalized to total protein concentration as assessed by the Bradford assay (Bio-Rad Laboratories). To visualize calcium deposition, VSMCs were fixed with 4% paraformaldehyde and stained with 2% Alizarin Red (pH4.5). The calcified areas are shown as red staining.

### Alkaline phosphatase (ALPL) activity assay

VSMCs were washed with PBS and assayed for ALPL activity using the ALPL colorimetric assay kit (Abcam) according to the manufacturer’s protocol. ALPL activity was normalized to total protein concentration as assessed by the Bradford assay (Bio-Rad Laboratories).

### Luciferase assay

HAoSMCs were transfected for 48 hours with 1 µg DNA mixture of MRE/GRE-responsive luciferase construct and a constitutively expressing *Renilla* construct (40:1 ratio, Qiagen) using X-tremeGENE HP DNA transfection reagent (Roche Applied Science) according to the manufacturer’s protocol. *Renilla-*Luciferase served as control for transfection efficiency. HAoSMCs were silenced and/or treated as indicated. After the incubation period, cells were washed with PBS, lysed with Passive Lysis Buffer (Promega) and assayed for transcriptional activity using Dual-Luciferase Reporter Assay (Promega) and a luminometer (Walter Wallac 2 plate reader, Perkin Elmer) according to the manufacturer’s protocol. All results are expressed as the ratio of MRE/GRE Firefly-Luciferase to *Renilla*-Luciferase (relative light units) normalized to Neg.si silenced or control treated HAoSMCs.

### Steroid quantification

After the incubation period, the cell culture medium was stored at −80 °C. Steroid hormone levels [cortisol, cortisone, corticosterone, 11-dehydrocorticosterone, 11-deoxycorticosterone, aldosterone, dehydroepiandrosterone (DHEA), androstene-3,17-dione and testosterone] were determined as described previously with minor adaptations^63^. Briefly, for solid-phase extraction, each cell culture supernatant (4 mL) was mixed with protein precipitation solution (0.8 M zinc sulphate in water/methanol; 50/50, v/v, 1 mL) that contained deuterium-labeled aldosterone, corticosterone, androstenedione, and testosterone as internal standards (ISTD). Samples were incubated in a thermoshaker by thoroughly shaking (10 min at 4 °C, 1000 rotations/min). The samples were centrifuged (10 min at 4 °C, 3184 × g). The supernatants were transferred to Oasis HBL SPE cartridges (Waters, 60 mg), preconditioned with methanol and water. After washing twice with water (3 ml) and twice with methanol/water (3 ml, 10/90, v/v), the steroids were eluted with methanol (3 mL) and evaporated to dryness (35 °C). The samples were reconstituted in methanol (50 µL). All steroids were separated and quantified by ultra-pressure LC-MS/MS (UPLC-MS/MS) using an Agilent 1290 UPLC coupled to an Agilent 6490 triple quadrupole mass spectrometer equipped with a jet-stream electrospray ionization interface (Agilent Technologies). Analyte separation was achieved using a reverse-phase column (1.7 µm, 150 mm; Acquity UPLC BEH C18; Waters). MASSHUNTER software (Agilent Technologies) was used for data acquisition and analysis. The accuracy was between 85% and 115% for all analytes.

### Quantitative RT-PCR

Quantitative RT-PCR was performed as described^20^. Total RNA was isolated from mouse tissue and VSMCs by using Trifast Reagent (Peqlab) according to the manufacturer’s instructions. Reverse transcription of 2 µg RNA was performed using oligo(dT)_12–18_ primers (Thermo Fisher Scientific) and SuperScript III Reverse Transcriptase (Thermo Fisher Scientific). Quantitative RT-PCR was performed with the iCycler iQ^TM^ Real-Time PCR Detection System (Bio-Rad Laboratories) and iQ^TM^ Sybr Green Supermix (Bio-Rad Laboratories) according to the manufacturer’s instructions. A detailed description of the primers is available in the supplementary material. The specificity of the PCR products was confirmed by analysis of the melting curves. All PCRs were performed in duplicate and relative mRNA fold changes were calculated by the 2^−ΔΔCt^ method using Gapdh as internal reference.

### Extraction of nuclear and cytoplasmic proteins

The preparation of cytoplasmic and nuclear extracts from HAoSMCs was performed using the NE-PER nuclear and cytoplasmic extraction reagents (Thermo Fisher Scientific) according to the manufacturer’s instructions. Protein concentration was determined by Bradford assay (Biorad Laboratories).

### Western blot analysis

After washing with PBS, HAoSMCs were lysed with ice-cold IP lysis buffer (Thermo Fisher Scientific) supplemented with complete protease and phosphatase inhibitor cocktail (Thermo Fisher Scientific). After centrifugation at 10000 rpm for 5 min, the proteins were boiled in Roti-Load1 Buffer (Carl Roth GmbH) at 100 °C for 10 min. Proteins were separated on SDS-polyacrylamide gels and transferred to PVDF membranes. The membranes were incubated overnight at 4 °C with primary antibodies: rabbit anti-CYP11B2 (diluted 1:1000, Abcam), goat anti-APEX1 (diluted 1:500, Santa Cruz Biotechnology), rabbit anti-HDAC2, rabbit anti-α-Tubulin or rabbit anti-GAPDH antibody (diluted 1:1000, Cell Signaling) and then with secondary anti-goat HRP-conjugated (diluted 1:1000, Santa Cruz Biotechnology) or anti-rabbit HRP-conjugated antibody (diluted 1:1000, Cell Signaling) for 1 hour at RT. For loading controls, the membranes were stripped in stripping buffer (Thermo Fisher Scientific) at RT for 10 min. Antibody binding was detected with ECL detection reagent (Thermo Fisher Scientific). Bands were quantified with Quantity One Software (Bio-Rad Laboratories).

### Confocal microscopy

For immunohistochemistry, 4% paraformaldehyde-fixed murine thoracic aortic tissues were cryoprotected in 30% sucrose, frozen in mounting medium (Tissue-Tek, Sakura Finetek) and sectioned at a thickness of 8 µm on coated slides. For immunostaining, sections were dehydrated at RT for 30 min and fixed in 100% methanol for 10 min at RT. For immunocytochemistry, VSMCs cultured onto four-well chamber slides (BD Biostatus) were fixed with 4% paraformaldehyde/PBS for 15 min at RT and permeabilized with ice-cold 100% methanol for 10 min at RT. To reduce non-specific background staining, slides were incubated with 5% normal goat serum or with 5% BSA in PBS/ 0.1% Triton-X100 for 1 hour at RT. Sections were incubated overnight at 4 °C with primary antibodies: goat polyclonal anti-Cyp11b2, goat polyclonal anti-APEX1, rabbit polyclonal anti-Cbfa1 or goat polyclonal anti-Msx2 (diluted 1:50, Santa Cruz Biotechnology). Binding of primary antibodies was visualised using goat anti-rabbit Alexa488-conjugated antibody or donkey anti-goat Alexa488-conjugated antibody (diluted 1:1000, Thermo Fisher Scientific) incubated for 1 hour at RT. Nuclei were stained using DRAQ5 dye (diluted 1:1000, Biostatus) and actin using Rhodamine Phalloidin (diluted 1:100, Thermo Fisher Scientific). The slides were mounted with Prolong Gold antifade reagent (Thermo Fisher Scientific). Images were collected with a confocal laser-scanning microscope (LSM 510, Carl Zeiss MicroImaging GmbH) using a water immersion A-Plan 40x/1.2 W DICIII or A-Plan × 63/1.2 W. Confocal images are representative for four mice per group or four independent experiments, respectively. Negative controls were carried out simultaneously with all experiments by omitting incubation with primary antibodies.

### Human coronary arteries

The procedure was approved by the Ethical Committee of the Medical University of Graz (ref. No: 26-355-ex13/14) and was carried out in accordance with the Declaration of Helsinki. Coronary biopsies were obtained from brain dead multi organ donors where hearts were not suitable for transplantation (Supplementary Table S4, n = 19). Biopsies were transferred in Custodiol® Solution, (Dr. Franz Köhler Chemie GmbH) and subsequently dissected and immediately snap frozen in liquid nitrogen. Serum and plasma samples were centrifuged at 4 °C with 3000 rpm, aliquoted and snap frozen in liquid nitrogen. From 2 patients, no serum or plasma samples were obtainable. Samples were separated in two groups according to renal function as assessed by plasma creatinine levels. Creatinine levels below 1.1 (mg/dl) were defined as normal renal function (CTR, n = 10) at the time of assessment. The impaired renal function (IRF) group (n = 9) had elevated plasma creatinine (mean = 2.0 ± 0.2 mg/dl) levels. Aldosterone was measured in plasma using an ELISA kit (Alpha Diagnostics International) according to the manufacturer’s protocol. Plasma aldosterone levels (CTR: 222 ± 31 pg/ml; IRF: 303 ± 43 pg/ml, n = 8–9) or plasma phosphate levels (CTR: 3.2 ± 0.5 mg/dl; IRF: 4.0 ± 0.4 mg/dl, n = 8–9) did not differ significantly between the groups. However, due to the sample origin from brain dead multi organ donors these measurements should be interpreted with caution.

### Statistics

Data are shown as arithmetic mean ± SEM unless indicated otherwise. N indicates the number of independent experiments performed at different passages of the cells or the number of mice examined, respectively. Normality was tested with Shapiro-Wilk test. Non-normal datasets were transformed (log, reciprocal or sqrt) prior to statistical testing to provide normality according to Shapiro-Wilk test. Statistical testing was performed by one-way Anova followed by Tukey-test for homoscedastic data or Games-Howell test for heteroscedastic data. Non-normal data was tested by the Steel-Dwass method. Two groups were compared by unpaired two-tailed t-test. Results from human coronary arteries are shown as box-plots or scatter-plot of the log-transformed relative mRNA expression. For correlation analysis, Pearson correlation test was performed. p < 0.05 was considered statistically significant.

## Electronic supplementary material

Supplemental Material
