# Supplementary material for: Involvement Of Vascular Aldosterone Synthase In Phosphate-Induced Osteogenic Transformation Of Vascular Smooth Muscle Cells
Source: Sci Rep. 2017 May 17;7:2059. doi: 10.1038/s41598-017-01882-2 (PMC5435689; doi:10.1038/s41598-017-01882-2)

INVOLVEMENT OF VASCULAR ALDOSTERONE SYNTHASE IN PHOSPHATE-INDUCED OSTEOGENIC TRANSFORMATION OF VASCULAR SMOOTH MUSCLE CELLS

Ioana Alesutan[1,2,3], Jakob Voelkl[1,2], Martina Feger[1], Denise V. Kratschmar[4], Tatsiana Castor[1], Sobuj Mia[1], Michael Sacherer[5], Robert Viereck[1], Oliver Borst[1,6], Christina Leibrock[1], Meinrad Gawaz[6], Makoto Kuro-o[7], Stefan Pilz[8], Andreas Tomaschitz[5,9], Alex Odermatt[4], Burkert Pieske[2,3,10], Carsten A. Wagner[11], Florian Lang[1*]

[1]Department of Physiology, University of Tübingen, Tübingen, Germany; [2]Department of Internal Medicine and Cardiology, Charité University Medicine, Campus Virchow-Klinikum, Berlin, Germany; [3]Berlin Institute of Health (BIH), Berlin, Germany; [4]Department of Pharmaceutical Sciences, and the National Center for Excellence in Research NCCR Kidney, University of Basel, Basel, Switzerland; [5]Div. of Cardiology, Medical University of Graz and Ludwig Boltzmann Institute for Translational Heart Failure Research, Graz, Austria; [6]Department of Cardiology and Cardiovascular Medicine, University of Tübingen, Tübingen, Germany; [7]Center for Molecular Medicine, Jichi Medical University, Japan; [8]Department of Internal Medicine, Division of Endocrinology and Metabolism, Medical University of Graz, Graz, Austria; [9]Bad Gleichenberg Clinic, Bad Gleichenberg, Austria; [10]Department of Cardiology, University of Graz, Graz, Austria; Department of Internal Medicine and Cardiology, German Heart Center Berlin (DHZB), Berlin, Germany; [11]Institute of Physiology, University of Zurich, and the National Center for Excellence in Research NCCR Kidney, Zurich, Switzerland.

Supplemental material

Supplemental Methods

Quantitative RT-PCR
The following mouse primers were used (5'→3' orientation):
Acta2 fw: CCCAGACATCAGGGAGTAATGG;
Acta2 rev: TCTATCGGATACTTCAGCGTCA;
Alpl fw: TTGTGCCAGAGAAAGAGAGAGA;
Alpl rev: GTTTCAGGGCATTTTTCAAGGT;
Cyp11b2 fw: TATCCATGCCCTGCATTCTATGT;
Cyp11b2 rev: AGTCAAGCTCTTGGGTAAGAACA;
Cyp11b2 (intron-spanning) fw: ACTCGGGTGTGGAAAGAACAT;
Cyp11b2 (intron-spanning) rev: GCCACTGTAGGTCTGAGAGC;
Cbfa1 fw: AGAGTCAGATTACAGATCCCAGG;
Cbfa1 rev: AGGAGGGGTAAGACTGGTCATA;
Gapdh fw: AGGTCGGTGTGAACGGATTTG;
Gapdh rev: TGTAGACCATGTAGTTGAGGTCA;
Msx2 fw: TTCACCACATCCCAGCTTCTA;
Msx2 rev: TTGCAGTCTTTTCGCCTTAGC;
Pit1 fw: TTTGACAAACTTCCTCTGTGGG;
Pit1 rev: GGACTTTCAGACGGACTAGACTT.
The following human primers were used (5'→3' orientation):
ACE fw: AACATGCAAATAGCCAACCACA;
ACE rev: TGCCCGTTCTAGGTCCTGAA;
ACTA2 fw: AAAAGACAGCTACGTGGGTGA;
ACTA2 rev: GCCATGTTCTATCGGGTACTTC;
AGTR1 fw: GCCCTTTGGCAATTACCTATGT;
AGTR1 rev: CGTGAGTAGAAACACACTAGCGT;
AGTR2 fw: GTACCAATCTGTCATCTACCCCT;
AGTR2 rev: CAGGCCATACACCAAACAAGG;
ALPL fw: GGGACTGGTACTCAGACAACG;
ALPL rev: GTAGGCGATGTCCTTACAGCC;
APEX1 fw: GTTTCTTACGGCATAGGCGAT;
APEX1 rev: CACAAACGAGTCAAATTCAGCC;
ATF2 fw: AATTGAGGAGCCTTCTGTTGTAG;
ATF2 rev: CATCACTGGTAGTAGACTCTGGG;
ATP6AP2 fw: AAATTGGCCTATACCAGGAGAGC;
ATP6AP2 rev: ATGAAACAGGTTACCCACTGC;
CYP11A1 fw: GAGGCCCAGCGATTCATTGAT;
CYP11A1 rev: TCCTGAACAGACGGAACAGGT;
CYP11B1 fw: GGGTGGCCTACAGACAACATC;
CYP11B1 rev: GGCGACAGCACTTCTGGATT;
CYP11B2 fw: TTCAACCGCCCTCAACACTAC;
CYP11B2 rev: GGAAACGCTGTCGTGTCCA;
CYP21A2 fw: CTCACCTTCGGAGACAAGATCA;
CYP21A2 rev: TCCACAATTTGGATGGACCAG;
CBFA1 fw: GCCTTCCACTCTCAGTAAGAAGA;
CBFA1 rev: GCCTGGGGTCTGAAAAAGGG;
GAPDH fw: GAGTCAACGGATTTGGTCGT;
GAPDH rev: GACAAGCTTCCCGTTCTCAG;
HSD3B1 fw: CACATGGCCCGCTCCATAC;
HSD3B1 rev: GTGCCGCCGTTTTTCAGATTC;
HSD3B2 fw: CTTGTGCGTTAAGACCCACAT;

HSD3B2 rev: GGGTTGACTGTAGAGAACTTTCC;
NR3C1 fw: ATAGCTCTGTTCCAGACTCAACT;
NR3C1 rev: TCCTGAAACCTGGTATTGCCT;
NR3C2 fw: AGCAGAACCAACAAGGAAGCA;
NR3C2 rev: GTGTTCACACAACTTAGAGTGGA;
PiT1 fw: GGAAGGGCTTGATTGACGTG;
PiT1 rev: CAGAACCAAACATAGCACTGACT;
SOX9 fw: AGCGAACGCACATCAAGAC;
SOX9 rev: CTGTAGGCGATCTGTTGGGG;
SP7 fw: CACAAAGAAGCCGTACTCTGT;
SP7 rev: GGGGCTGGATAAGCATCCC;
STARD1 fw: GGGAGTGGAACCCCAATGTC;
STARD1 rev: CCAGCTCGTGAGTAATGAATGT.

Supplemental Tables

Suppl. Table S1. Characteristics of WT and *kl/kl* mice treated with spironolactone or adrenalectomy for 5 weeks. Arithmetic means ± SEM of body weight (Bw; g), tail cuff systolic blood pressure (BP; mmHg), plasma concentration of calcium (mg/dl) and phosphate (mg/dl) of wild-type (WT) and *kl/kl* mice following discontinuation of dietary rescue and without or with treatment with spironolactone (Spr) or adrenalectomy (Adx) for 5 weeks.

	**WT**	**WT-Adx**	***kl/kl***	***kl/kl*-Spr**	***kl/kl*-Adx**	
Bw [g]	24.2 ± 0.6	22.7 ± 0.7	24.2 ± 0.8	22.5 ± 0.9	23.0 ± 0.7	n=10-11
Tail cuff sys. BP [mmHg]	101.1 ± 2.2	98.5 ± 6.2	109.1 ± 6.9	99.6 ± 4.9	102.4 ± 6.2	n=5-9
Calcium [mg/dl]	8.6 ± 0.1	9.0 ± 0.2	9.5 ± 0.2 *	9.6 ± 0.2 *	9.6 ± 0.3 *	n=9
Phosphate [mg/dl]	7.5 ± 0.3	7.9 ± 0.4	9.6 ± 0.4 *	10.7 ± 0.4 **	10.2 ± 0.5 **	n=9

Suppl. Table S2. Characteristics of WT and *kl/kl* mice treated with spironolactone and adrenalectomy for 5 weeks. Arithmetic means ± SEM of body weight (Bw; g), tail cuff systolic blood pressure (BP; mmHg), plasma concentrations of calcium (mg/dl) and phosphate (mg/dl) of wild-type (WT) and *kl/kl* mice following discontinuation of dietary rescue and without or with adrenalectomy treatment (Adx) with or without treatment with spironolactone (Spr) for 5 weeks.

	**WT**	**WT-Adx**	***kl/kl*-Adx**	***kl/kl*-Adx+Spr**	
Bw [g]	26.7 ± 0.8	23.8 ± 1.0	22.1 ± 0.7 **	21.8 ± 0.6 ***	n=10-11
Tail cuff sys. BP [mmHg]	107.8 ± 3.1	109.1 ± 4.7	104.8 ± 4.3	102.2 ± 2.9	n=8-10
Calcium [mg/dl]	8.7 ± 0.1	9 ± 0.2	9.5 ± 0.3 *	9.2 ± 0.1 *	n=9
Phosphate [mg/dl]	7.2 ± 0.4	8.3 ± 0.4	10.8 ± 0.5 ***	9.9 ± 0.7 **	n=9

Suppl. Table S3. Characteristics of patients with maintained and impaired renal function.

**Gender**	**Age**	**Creatinine [mg/dl]**	**Diabetes**	**BMI**	**Medication**
female	66	0.86	unknown	23	Desmopressine
female	60	0.71	N	25	Furosemide
male	66	0.73	N	25	Pantoprazole, Lisinopril, Fluoxetine, Nebivolol, Amlopidine, Trazodon hydrochloride, Paracetamole
male	46	0.69	N	28	Pantoprazole, Heparin, Urapidil, Desmopressine
male	77	1.03	N	27	Simvastatin, Furosemide, Eprosartan, Levothyroxine, Allopurinol
female	62	0.46	N	25	Acetylsalicylic acid , Metoprolol, Carbamazepine, Clonazepam, Simvastatin, Donezepil, Alendronate, Calcium D3, Pantoprazole
male	76	0.82	N	25	-
male	46	1.03	N	25	Levothyroxine
female	62	0.98	N	24	Zystec, Mefenaminacid
female	50	0.72	N	24	Acetylsalicylic acid
male	78	↑ **1.67**	Y	24	Digoxin, Nebivolol, Acenocoumarol, Sultamicillin, Sulbactam, Ampicillin, Insulin
male	73	↑ **1.53**	Y	24	Digoxin, Albumin, Sultamicillin, Insulin
male	56	↑ **1.51**	N	28	-
male	55	↑ **2.54**	N	26	Simvastatin, Cortisone, Midazolam hydrochloride, Desmopressine, Moxonidine, Amlopidine, Budesonide, Formaterol, Rilmenidine, Alprazolam
male	75	↑ **3,60**	Y	29	Enalapril, Metformin, Nocorandil, Moxonidine, Diphenhydramine, Chlorotheophylline
female	29	↑ **2.35**	unknown	21	Pantoprazole, Sufentanil, Metoclopramide, Desmopressine
male	51	↑ **1.49**	N	23	Phenprocoumon, Enoxaparin sodium, Ramipril, Diclofenac sodium, Paracetamole
male	74	↑ **1.59**	N	24	-
male	48	↑ **1.70**	N	29	Paracetamole, Pantoprazole

Supplemental Figures

Suppl. Fig. S1. Effect of mineralocorticoid receptor blockade on phosphate-induced osteo-/chondrogenic markers expression *in vitro.* Arithmetic means ± SEM of *SOX9* (**a**) and *SP7* (**b**) relative mRNA expression (n=8; arbitrary units, a.u.) in HAoSMCs following treatment with (Pi) or without (Ctr) phosphate and with or without additional treatment with 10µM spironolactone (Spr) or 10µM eplerenone (Epl). *(p<0.05), ***(p<0.001) statistically significant vs control treated HAoSMCs; †††(p<0.001) statistically significant vs Pi treated HAoSMCs.

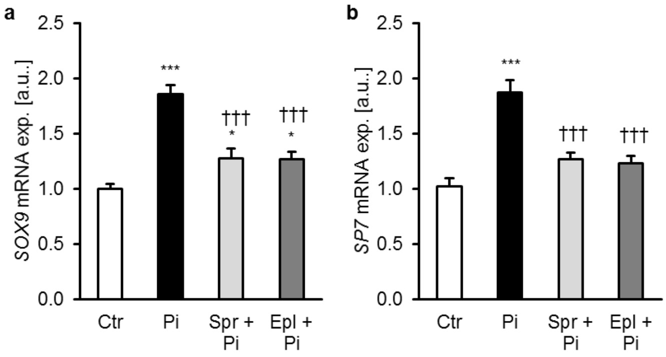

Suppl. Fig. S2. NR3C2 silencing efficiency in HAoSMCs. Arithmetic means ± SEM (n=10; arbitrary units, a.u.) of *NR3C2* relative mRNA expression in HAoSMCs silenced for 48 hours with negative control siRNA (Neg.si.) or with MR siRNA (MRsi.). ***(p<0.001) statistically significant vs. Neg.si. silenced HAoSMCs.

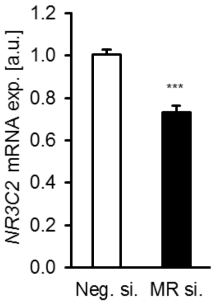

Suppl. Fig. S3. Expression of aldosterone synthesis pathway enzymes in H295 cells and HAoSMCs. a. Representative original bands and arithmetic means ± SEM (n=4; arbitrary units, a.u.) of *CYP11B2* relative mRNA expression in H295 cells and in HAoSMCs (NTC– no template control; N.D. - not detected). **b.** Arithmetic means ± SEM (n=4; a.u.) of *CYP11B2*, *CYP11A1*, *HSD3B1*, *HSD3B2*, *CYP21A2*, *STARD1* and *CYP11B1* relative mRNA expression in H295 cells (black bars) and in HAoSMCs (white bars).

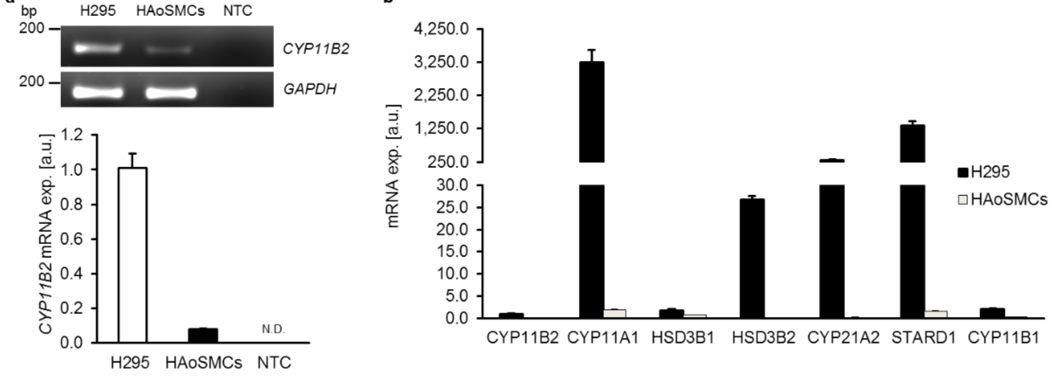

Suppl. Fig. S4. Effect of phosphate on expression of aldosterone synthesis pathway enzymes in HAoSMCs. Arithmetic means ± SEM (n=4; arbitrary units, a.u.) of *CYP11A1* (**a**), *HSD3B1* (**b**), *HSD3B2* (**c**), *CYP21A2* (**d**), *STARD1* (**e**) and *CYP11B1* (**f**) relative mRNA expression in H295 cells and in HAoSMCs following treatment for 24 hours with (Pi) or without (Ctr) phosphate. *($p<0.05$) statistically significant vs. control treated HAoSMCs (t-test).

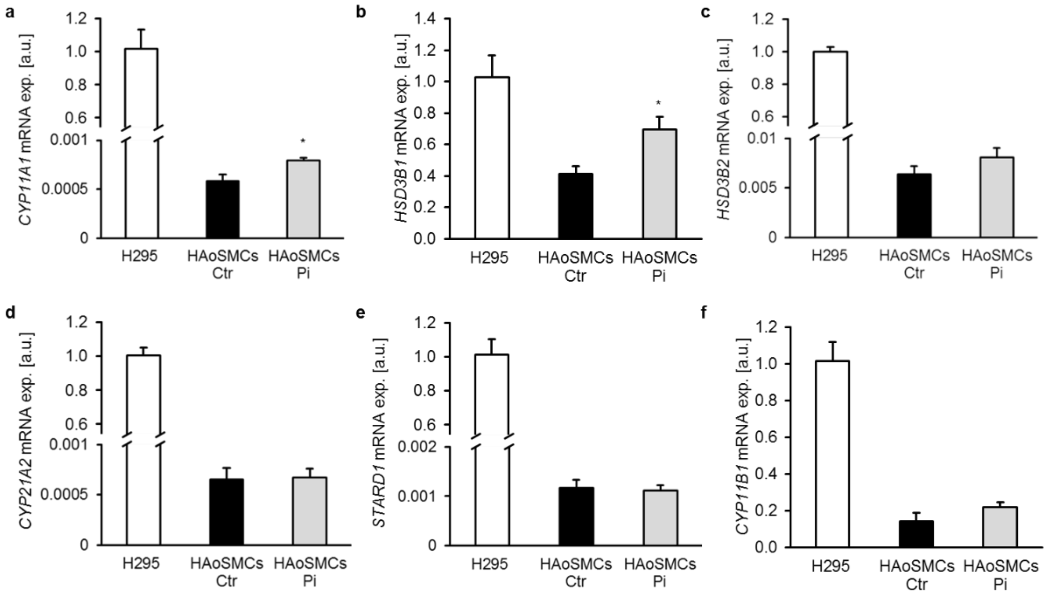

Suppl. Fig. S5. Effect of phosphate on expression of renin-angiotensin system in HAoSMCs. Arithmetic means ± SEM (n=6; arbitrary units, a.u.) of *ATP6AP2* (**a**), *ACE* (**b**), *AGTR1* (**c**) and *AGTR2* (**d**) relative mRNA expression in HAoSMCs following treatment for 24 hours with (Pi) or without (Ctr) phosphate.

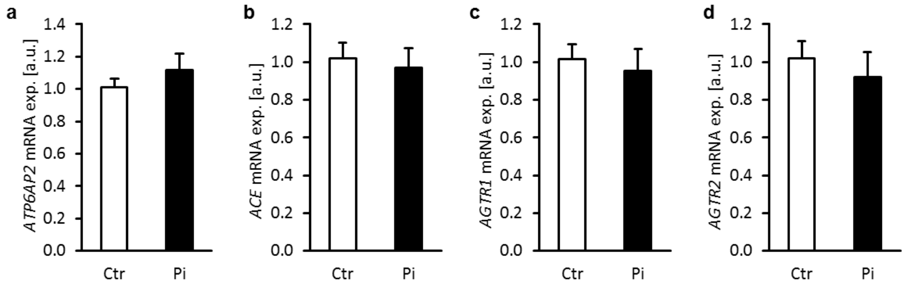

Suppl. Fig. S6. Effect of angiotensin II and phosphate on aldosterone synthase mRNA expression in HAoSMCs. Arithmetic means ± SEM (n=6; arbitrary units, a.u.) of *CYP11B2* relative mRNA expression in HAoSMCs following treatment for 24 hours with control (Ctr), angiotensin II (AngII), phosphate (Pi) or angiotensin II together with phosphate. ***(p<0.001) statistically significant vs. control treated HAoSMCs.

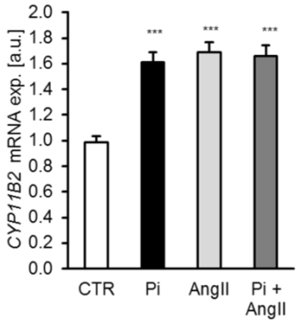

Suppl. Fig. S7. Effect of phosphate on *NR3C2* and *NR3C1* mRNA expression in HAoSMCs. Arithmetic means ± SEM (n=6; arbitrary units, a.u.) of *NR3C2* (**a**) and *NR3C1* (**b**) relative mRNA expression in HAoSMCs following treatment for 24 hours with (Pi) or without (Ctr) phosphate.

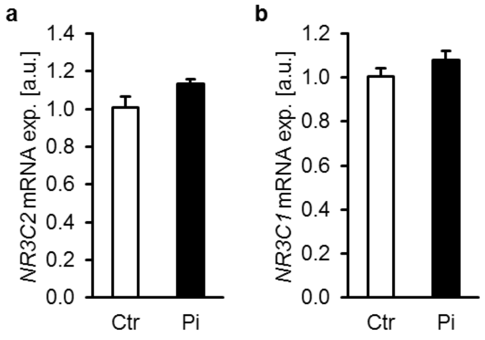

Suppl. Fig. S8. *Cyp11b2* mRNA expression in *kl/kl* mice and in MAoSMCs. a. Arithmetic means ± SEM (n=8; arbitrary units, a.u.) of *Cyp11b2* relative mRNA expression (intron-spanning primers) in aortic tissue from *kl/kl* mice and corresponding wild-type mice (WT). **b.** Arithmetic means ± SEM (n=9, a.u.) of *Cyp11b2* relative mRNA expression (intron-spanning primers) in MAoSMCs isolated from wild-type mice (Cyp11b2$^{+/+}$) treated for 24 hours with (Pi) or without (Ctr) phosphate. **(p<0.01) statistically significant vs. WT mice or control treated MAoSMCs, respectively.

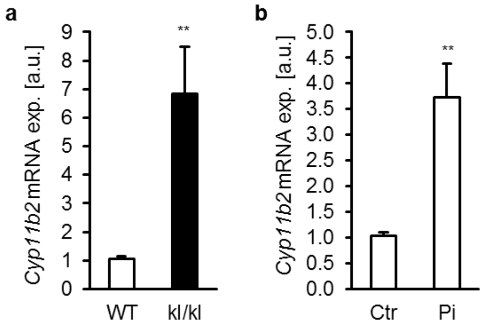

Suppl. Fig. S9. Quantification of steroids in HAoSMCs culture medium. Cell culture medium of HAoSMCs (1mL) from four independent samples per condition (control and Pi (2mM) incubated for either 24 or 48 hours), was pooled to a final sample volume of 4mL and underwent sample preparation for LC-MS/MS analysis. Steroids were measured and quantified as described in methods section (**Table**; ND = not detected, NQ = not quantified). Representative data of aldosterone with aldosterone-D7 as internal standard (ISTD, dotted line) (**a**) and cortisone (**b**) measured by LC-MS/MS in the HAoSMCs culture medium (4mL) treated for 48 hours with (Pi, Solid line) or without (CTR, Dashed line) phosphate.

	Aldosterone [pM]	Cortisone [pM]	Cortisol [pM]	11-Dehydrocorticosterone [pM]	Corticosterone [pM]	11-Deoxycorticosterone [pM]	Androstenedione [pM]	Testosterone [pM]	DHEA [pM]
CTR (24h)	ND	22.7	ND	NQ	ND	19.7	14.7	31.1	ND
Pi (24h)	ND	28.7	ND	NQ	ND	ND	23.6	61.1	ND
CTR (48h)	ND	24.1	ND	NQ	ND	ND	NQ	28.0	ND
Pi (48h)	ND	68.9	ND	34.8	ND	ND	12.9	79.3	1417.7

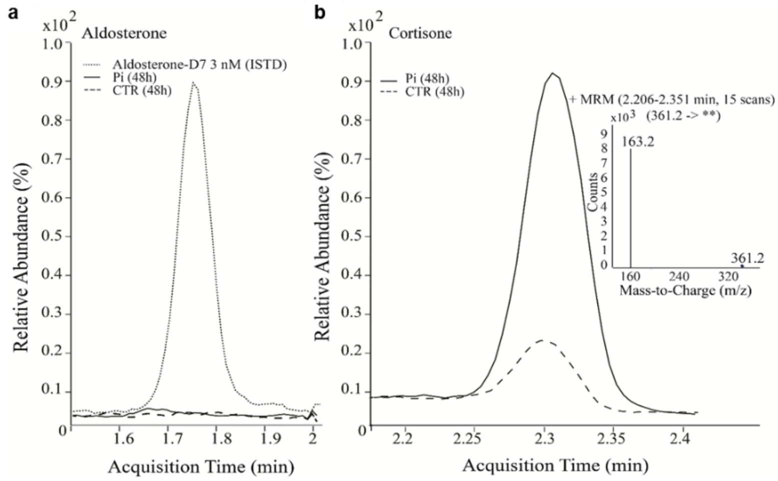

Suppl. Fig. S10. CYP11B2 silencing efficiency in HAoSMCs. a. Arithmetic means ± SEM (n=10; arbitrary units; a.u.) of *CYP11B2* relative mRNA expression in HAoSMCs silenced for 48 hours with negative control siRNA (Neg.si.) or with CYP11B2 siRNA (C11B2si.). **b**. Representative original Western blots and arithmetic means ± SEM (n=8; a.u.) of normalized CYP11B2/GAPDH protein ratio in HAoSMCs silenced for 48 hours with negative control siRNA (Neg.si.) or with CYP11B2 siRNA (C11B2si.). **c.** Arithmetic means ± SEM (n=4; a.u.) of MRE/GRE-dependent transcriptional activity measured by luciferase reporter assay in HAoSMCs silenced for 48 hours with negative control siRNA (Neg.si.) or with CYP11B2 siRNA (C11B2si.) and treated for 24 hours with (Pi) or without (Ctr) phosphate. *(p<0.05), **(p<0.01), ***(p<0.001) statistically significant vs. Neg.si. silenced HAoSMCs; †(p<0.05) statistically significant vs. Pi treated Neg.si. silenced HAoSMCs.

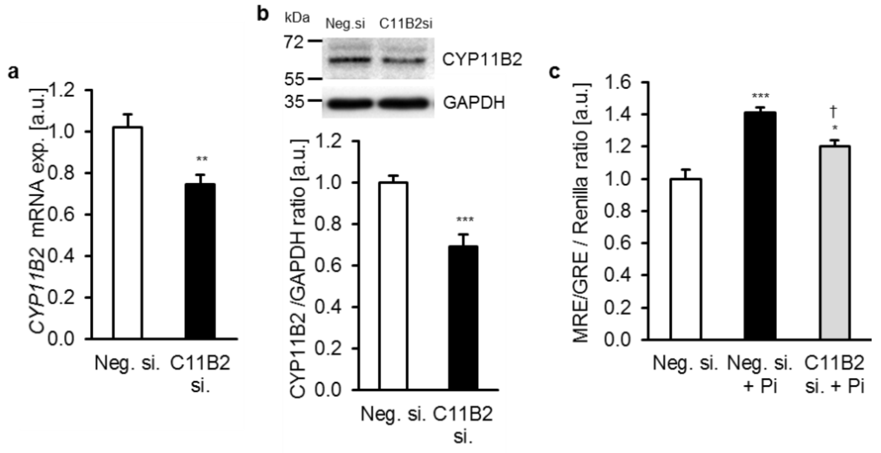

Suppl. Fig. S11. Effect of aldosterone synthase inhibition on phosphate-induced *ACTA2* mRNA expression *in vitro*. a. Arithmetic means ± SEM (n=6, arbitrary units, a.u.) of *ACTA2* relative mRNA expression in HAoSMCs silenced with negative control siRNA (Neg.si.) or CYP11B2 siRNA (C11B2si.) and treated with (Pi) or without (Ctr) phosphate and with or without additional treatment with 100nM aldosterone (Aldo). ***(p<0.001) statistically significant vs. Neg.si. silenced HAoSMCs. ††(p<0.01) statistically significant vs. Pi treated Neg.si. silenced HAoSMCs; §§§(p<0.001) statistically significant between Pi and Pi+Aldo treated C11B2si silenced HAoSMCs. **b.** Arithmetic means ± SEM (n=9, a.u.) of *Acta2* relative mRNA expression in MAoSMCs isolated from aldosterone synthase-deficient mice ($Cyp11b2^{-/-}$) or corresponding wild-type mice ($Cyp11b2^{+/+}$) treated with (Pi) or without (Ctr) phosphate and with or without additional treatment with 100nM aldosterone (Aldo). *(p<0.05) statistically significant vs. respective control treated MAoSMCs.

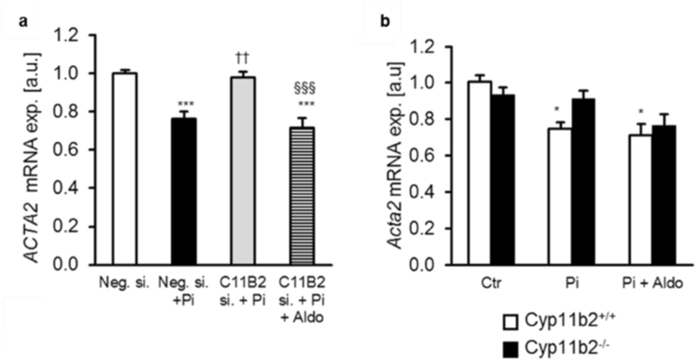

Suppl. Fig. S12. Effect of phosphate on APEX1 expression in HAoSMCs. a. Arithmetic means ± SEM (n=6; arbitrary units, a.u.) of *APEX1* relative mRNA expression in HAoSMCs following treatment for 24 hours with (Pi) or without (Ctr) phosphate. **b**. Representative original Western blots and arithmetic means ± SEM (n=4; a.u.) of normalized APEX1/GAPDH protein ratio in HAoSMCs following treatment for 24 hours with (Pi) or without (Ctr) phosphate. **c.** Arithmetic means ± SEM (n=8; a.u.) of *APEX1* relative mRNA expression in HAoSMCs silenced for 48 hours with negative control siRNA (Neg.si.) or with APEX1 siRNA (APEX1si.). ***(p<0.001) statistically significant vs. control treated or Neg.si. silenced HAoSMCs, respectively.

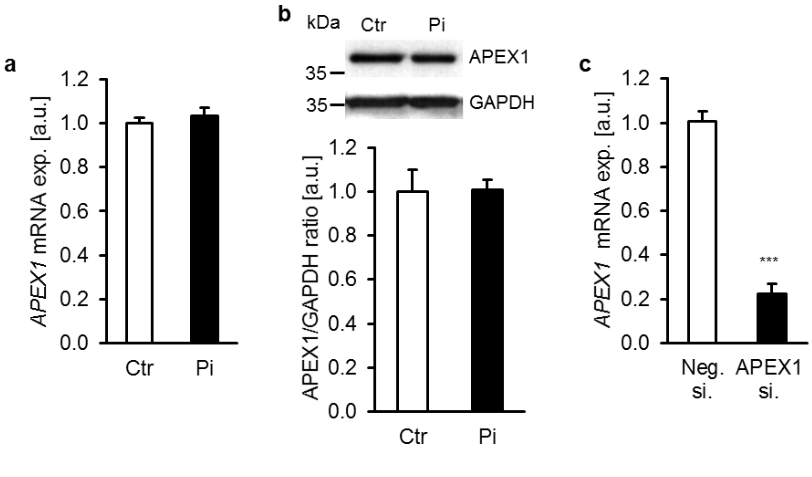

Suppl. Fig. S13. Effect of ATF2 silencing on *CYP11B2* mRNA expression in HAoSMCs. Arithmetic means ± SEM (n=6; arbitrary units, a.u.) of *ATF2* (**a**) and *CYP11B2* (**b**) relative mRNA expression in HAoSMCs following silencing for 48 hours with negative control siRNA (Neg.si.) or ATF2 siRNA (ATF2si.) without or with treatment for 24 hours with phosphate (Pi). **(p<0.01), ***(p<0.001) statistically significant vs. Neg.si. silenced HAoSMCs.

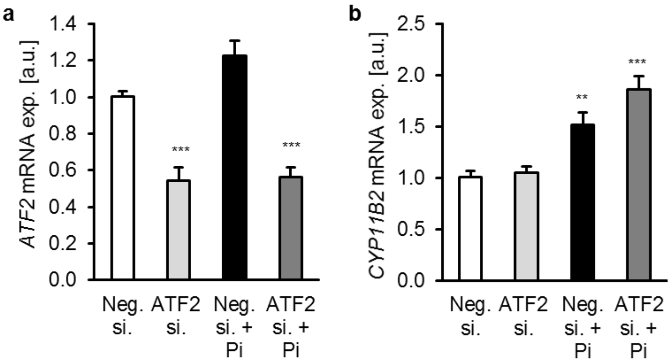

Suppl. Fig. S14. APEX1 transfection efficiency in HAoSMCs. Arithmetic means ± SEM of *APEX1* relative mRNA expression (**a**, n=8; arbitrary units, a.u.), calcium content (**b**, n=4; µg/mg protein) and alkaline phosphatase activity (**c**, n=4; units/mg protein) in HAoSMCs transfected for the period described in methods section with empty vector (V) or with human APEX1 (APEX1). **(p<0.01) statistically significant vs. empty vector transfected HAoSMCs.

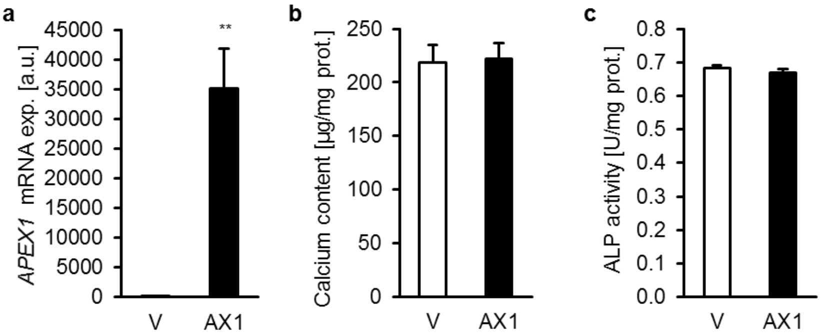

Suppl. Fig. S15. Vascular osteoinductive transformation is ameliorated by spironolactone, but not by adrenalectomy in *kl/kl* mice. Arithmetic means ± SEM (n=10-11; arbitrary units, a.u.) of aortic *Cyp11b2* (**a**) and *Pit1*, *Cbfa1*, *Msx2* and *Alpl* (**b**) relative mRNA expression in wild-type (WT) and *kl/kl* mice following discontinuation of dietary rescue and treatment without or with spironolactone (Spr) or adrenalectomy (Adx) for 5 weeks. *(p<0.05), **(p<0.01), ***(p<0.001) statistically significant vs. WT control mice. †(p<0.05), ††(p<0.01) statistically significant vs. *kl/kl* control mice, §(p<0.05), §§(p<0.01) statistically significant between *kl/kl*-Adx and *kl/kl*-Spr mice. **c.** Representative confocal microscopy images showing Cyp11b2, Cbfa1 and Msx2 protein expression in aortic tissue from wild-type (WT) and *kl/kl* mice following discontinuation of dietary rescue and treatment without or with spironolactone (Spr) or adrenalectomy (Adx) for 5 weeks. Images are representative of four mice per group. Protein expression: green labeling; nuclei: blue labeling and actin staining: red labeling. Scale bar: 20 µm.

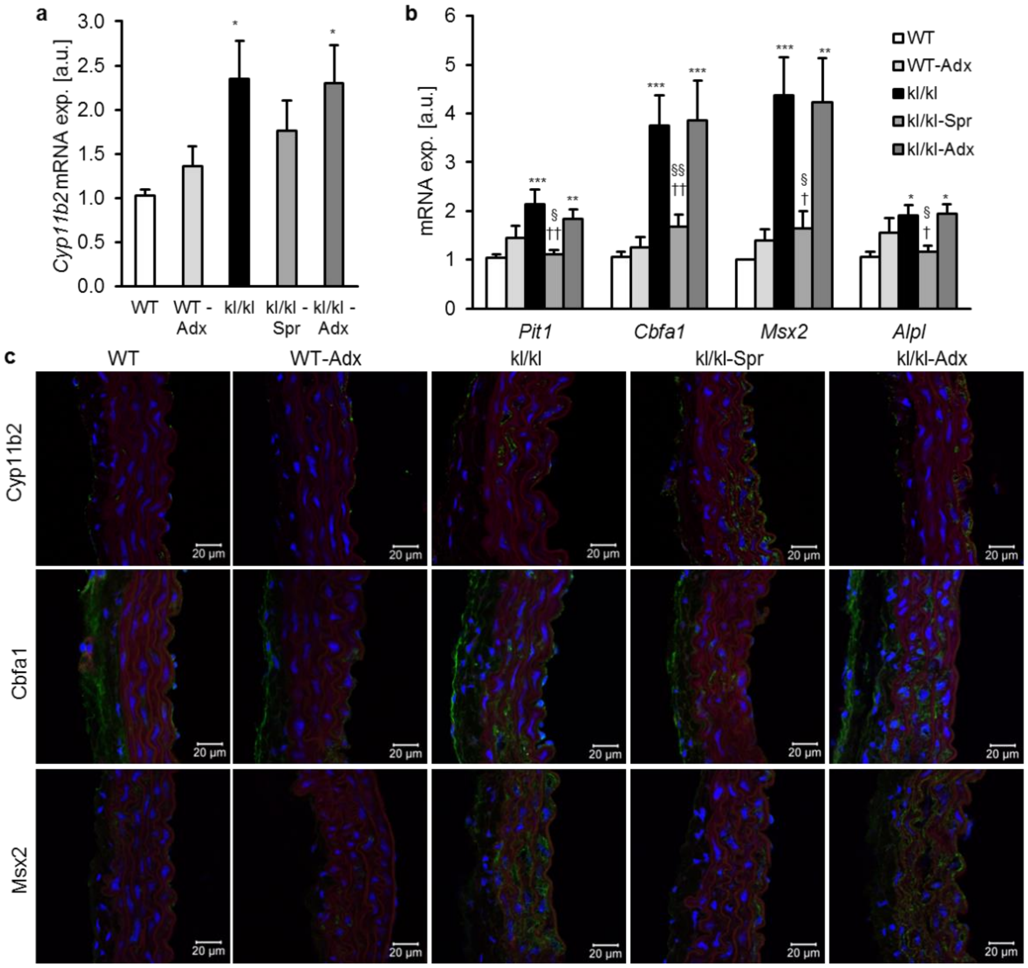

Supplement: Supplementary file 1 — Supplemental Material [file 41598_2017_1882_MOESM1_ESM.pdf]
